# Escherichia coli Uses a Dedicated Importer and Desulfidase To Ferment Cysteine

**DOI:** 10.1128/mbio.02965-21

**Published:** 2022-04-04

**Authors:** Yidan Zhou, James A. Imlay

**Affiliations:** a Department of Microbiology, University of Illinois, Urbana, Illinois, USA; Michigan State University; New York University School of Medicine

**Keywords:** amino acid fermentation, cysteine import, cysteine desulfidase, Crp, *cyuA*, *yhaO*, *yhaM*, DecR, CyuP, *cyuR*

## Abstract

CyuA of Escherichia coli is an inducible desulfidase that degrades cysteine to pyruvate, ammonium, and hydrogen sulfide. Workers have conjectured that its role may be to defend bacteria against the toxic effects of cysteine. However, *cyuA* sits in an operon alongside *cyuP*, which encodes a cysteine importer that seems ill suited to protecting the cell from environmental cysteine. In this study, transport measurements established that CyuP is a cysteine-specific, high-flux importer. The concerted action of CyuP and CyuA allowed anaerobic E. coli to employ cysteine as either the sole nitrogen or the sole carbon/energy source. CyuA was essential for this function, and although other transporters can slowly bring cysteine into the cell, CyuP-proficient cells outcompeted *cyuP* mutants. Cells immediately consumed the ammonia and pyruvate that CyuA generated, with little or none escaping from the cell. The expression of the *cyuPA* operon depended upon both CyuR, a cysteine-activated transcriptional activator, and Crp. This control is consistent with its catabolic function. In fact, the *cyuPA* operon sits immediately downstream of the *thrABCDEFG* operon, which allows the analogous fermentation of serine and threonine; this arrangement suggests that this gene cluster may have moved jointly through the anaerobic biota, providing E. coli with the ability to ferment a limited set of amino acids. Interestingly, both the *cyu-* and *thr*-encoded pathways depend upon oxygen-sensitive enzymes and cannot contribute to amino acid catabolism in oxic environments.

## INTRODUCTION

Bacteria have a remarkable ability to exploit amino acids that they encounter in their habitats. High-affinity importers enable cells to scavenge even trace amounts in order to support protein translation, thereby sparing the energy and material involved in amino acid synthesis. When available in higher concentrations, environmental amino acids can also be broken down to serve as carbon or nitrogen sources. These assimilatory and catabolic processes have been detailed in many microbes and particularly in the model bacterium Escherichia coli. Importers have been identified for all but 1 of the 20 conventional amino acids.

The exception is cysteine. This amino acid is unusually chemically reactive and therefore has a special place in biology: proteins exploit its thiolate side chain in redox reactions, to coordinate metal cofactors, and as a potent nucleophile. However, this reactivity also destines it to participate in uncontrolled chemistry, both in the environment and within cells. In oxic habitats, extracellular cysteine is quickly oxidized to the disulfide species cystine (1). Accordingly, most microbes possess dedicated cystine importers that bring cystine into the cell, where it is quickly reduced to cysteine and assimilated into sulfur metabolism. Whenever its intracellular cysteine pool dwindles, E. coli induces both ATP- and proton motive force-driven cystine importers (TcyJLN and TcyP) (2).

However, workers have struggled to identify analogous importers of cysteine itself. Because E. coli is among the many bacteria that dwell in anoxic environments, it would be surprising if it never encountered extracellular cysteine. Yet no high-affinity cysteine importer has yet been identified and demonstrated, in this or any other bacterium.

That situation appears to be changing. A series of recent studies indicated that E. coli and related enterics express a cysteine transporter in concert with a cysteine-degrading desulfidase. The desulfidase itself was first discovered in Methanocaldococcus jannaschii, a thermophilic archaeon that was recovered from an underwater black smoker (3): cysteine → pyruvate + NH_4_^+^ + H_2_S (reaction 1). This desulfidase had no transporter partner, and it was suggested that its physiological purpose might be to route sulfur atoms from endogenous cysteine to the assembly of iron-sulfur clusters. However, a homolog was subsequently discovered in an In Vivo Expression Technology (IVET) selection for genes that the fish pathogen Yersinia ruckeri induces when it infects its host (4). Its desulfidase gene (*cyuA* here) is cotranscribed with a gene (*cyuP*) encoding an apparent importer. Because Y. ruckeri possesses the sulfide-independent Isc and Suf systems that build iron-sulfur clusters, the physiological role of the operon must be something else. Strikingly, mutants lacking *cyuA* were poorly virulent. (Note that CyuP also appears in the literature as YhaO; CyuA also is known as YhaM.)

The *cyuPA* operon was subsequently explored in Salmonella enterica serovar Typhimurium and E. coli. Its expression is controlled by the cysteine-sensing transcription factor CyuR (also known as YbaO and DecR), and *cyuPA* expression increases >50-fold when millimolar cysteine is added to culture medium (5, 6). *S.* Typhimurium has a second desulfidase, CdsH, which is also controlled by CyuR; in that bacterium, *cyuPA* is primarily induced under anoxic conditions, while *cdsH* is induced aerobically (6). E. coli lacks CdsH, and its *cyuPA* operon is induced by cysteine without regard to the oxygen level (1, 6).

In trying to understand the role of CyuP/CyuA, investigators noted that the same high doses of cysteine that induce *cyuPA* can also be toxic (5–7). Cysteine can enter cells as a pseudosubstrate of importers whose proper substrates are nonpolar amino acids; when this adventitious influx is large, cytoplasmic cysteine accumulates and can inhibit biosynthetic enzymes, disrupt metal metabolism, and drive oxidative stress (8–11). Mutants that lack CyuA are hypersensitive to this exogenous cysteine, prompting the hypothesis that the physiological role of CyuA is to protect these bacteria by degrading overlarge cysteine pools (6).

However, it seems unlikely that an enzyme that protects the cytoplasm from extracellular cysteine would be partnered with a protein that brings cysteine inside. In the present study, we found that CyuP and CyuA enable E. coli to grow anaerobically using cysteine as either its sole carbon or nitrogen source. Various features of these enzymes—their *K_M_* values, high flux capacities, and repression by better carbon sources—are in accord with this role. Interestingly, when E. coli is exposed to oxygen, CyuA is quickly poisoned, and the pathway fails. Indeed, *cyuPA* is found only in anaerobic and facultative microbes (3, 6). In E. coli, it is located adjacent to an operon that enables the fermentation of threonine and serine; thus, this genomic region allows this bacterium to use a limited set of amino acids as anaerobic carbon sources.

## RESULTS

### As reported, CyuA can protect E. coli from excess cysteine.

A *cyuP-lacZ* transcriptional fusion demonstrated that the *cyuPA* operon is induced when cells are exposed to high concentrations of cysteine but not to other amino acids (see Fig. S1 in the supplemental material). Induction depended on the transcriptional activator CyuR. These results match those reported by other groups in studies of *Yersinia*, Salmonella, and E. coli (4–7).

10.1128/mBio.02965-21.2 FIG S1 l-Cysteine specifically induces *cyuP-lacZ*. (A) The expression of *cyuP-lacZ* was determined in minimal glucose-ammonia medium to which the specified amino acids (5 mM) had been added. Cells were harvested after four generations of growth. Growth with l-cysteine was performed anaerobically to keep cysteine reduced. Note that cells were unable to grow with added valine or d-cysteine. (B) Induction is mediated by the CyuR transcription factor. The strains used are ZYD156 and ZYD177. Download FIG S1, TIF file, 1.3 MB. Copyright © 2022 Zhou and Imlay. 2022 Zhou and Imlay. https://creativecommons.org/licenses/by/4.0/ This content is distributed under the terms of the Creative Commons Attribution 4.0 International license.

**FIG 1:**
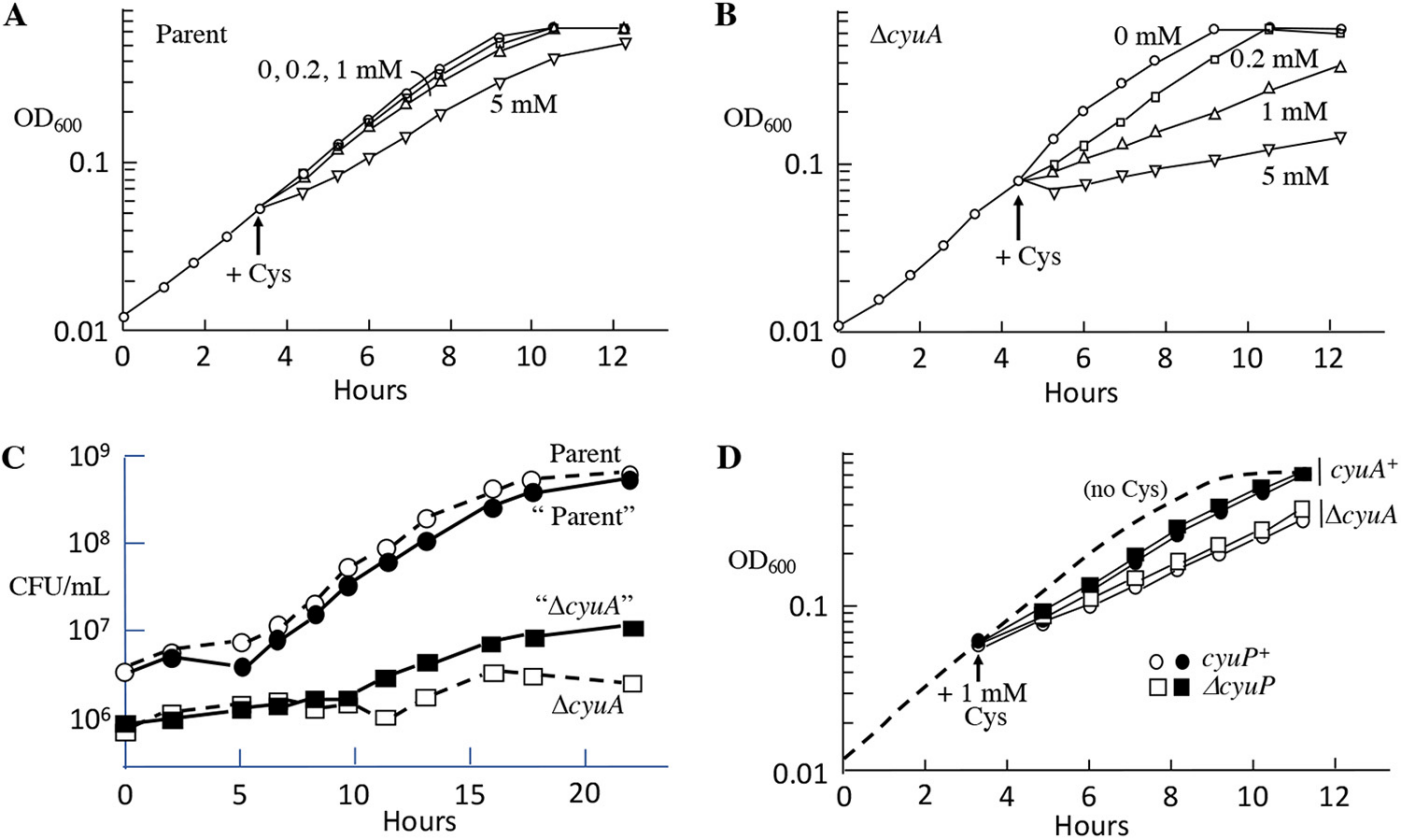
CyuA contributes to cysteine resistance, but CyuP does not. (A) At the arrow, cysteine was added to cells growing in anaerobic glucose-ammonium medium. The parent strain could adapt well to cysteine and showed only transient inhibition by 5 mM. The strain used is ZYD156. (B) Δ*cyuA* mutants exhibited greater cysteine sensitivity and no adaptation. The strain used is ZYD236. (C) CyuA protected cells by diminishing intracellular cysteine even with a limited impact on its extracellular concentration. Cysteine (5 mM) was added at time zero to the wild-type parent, a Δ*cyuA* mutant, and a mixture of the parent and mutant, all in glucose-ammonium medium. Cells were tracked by CFU; strains in the mixed culture are denoted by quotation marks. The parent in that culture outgrew the *cyuA* mutant. The strains used are KCI1266 and SSK195. (D) CyuP did not contribute to cysteine resistance. Cysteine (1 mM) was added at the arrow. The strains used are ZYD15, KCI1266, ZYD238, and SSK195.

Workers also reported that CyuA helps protect bacteria against high concentrations of cysteine (6, 7). We observed that 5 mM cysteine briefly impaired the growth of E. coli in defined medium; a lag was followed by restored growth, consistent with the induction of a defensive response (Fig. 1A). A *cyuA* mutant was more sensitive and did not exhibit a recovery (Fig. 1B).

Under the same conditions, the desulfidase activity of CyuA protected *cyuA^+^* cells more effectively than it did cocultured Δ*cyuA* mutants, indicating that its action was fast enough to lower the intracellular cysteine concentration substantially below that of the surrounding environment (Fig. 1C). This feature is consistent with the hypothesis (6, 7) that CyuA is a defensive enzyme that shields the cell from incoming cysteine.

However, in this scenario, it is more difficult to explain the role of CyuP, a membrane-bound transporter that has been proposed to be a cysteine importer. In our experiments, the toxicity of cysteine was diminished when Ile was supplied, indicating that CyuA helps to protect cytoplasmic threonine deaminase from competitive inhibition by cysteine (8) (Fig. S2). If the operon were defensive and the cytoplasm is the site of toxicity, it is difficult to imagine that the induction of a cysteine importer would be helpful. Indeed, during cysteine exposure, CyuP appeared not to contribute to cysteine resistance in either CyuA^+^ cells or CyuA^−^ mutants (Fig. 1D).

10.1128/mBio.02965-21.3 FIG S2 Cysteine toxicity occurs within the cytoplasm. Cells were grown in anaerobic minimal glucose-ammonia medium. At the arrow, 1 mM cysteine was added; where indicated, 0.5 mM Ile was added as well. The Δ*cyuA* cells were impaired by this level of cysteine, and this toxicity was substantially relieved by the addition of isoleucine. This suggested that toxicity resulted from the ability of cysteine to inhibit cytoplasmic threonine dehydrogenase, the first enzyme of the Ile biosynthetic pathway. The strains used are KCI1266 and SSK195. Download FIG S2, TIF file, 1.3 MB. Copyright © 2022 Zhou and Imlay. 2022 Zhou and Imlay. https://creativecommons.org/licenses/by/4.0/ This content is distributed under the terms of the Creative Commons Attribution 4.0 International license.

Furthermore, in a previous study, we made observations suggesting that CyuP can aggravate, rather than relieve, cytoplasmic cysteine toxicity (1). In those experiments, cells that overimported cystine rapidly reduced it to cysteine and suffered from its effects. Consequent growth inhibition could be partially relieved by the induction of AlaE, an exporter that pumped cysteine back out of the cytoplasm, but this protective effect was optimal only when *cyuP* was deleted. We inferred that CyuP worsened the situation by reimporting the expelled cysteine. Collectively, these data suggest that the primary role of *cyuPA* is not to protect cells from excess cysteine.

### CyuP is a cysteine importer.

Experiments in *Y. ruckeri* indicated that *cyuP* mutants were ca. 50% slower at cysteine uptake, supporting the idea that CyuP is an importer (4). To test transport more directly, we induced CyuP by preculturing cells with cysteine, and we used a strain that lacked other transporters that might interfere with the experiment. We eliminated the LIV system, CycA, BrnQ, and YaaJ transporters; these are dedicated importers of branched-chain amino acids and alanine, but they also exhibit some adventitious activity with cysteine (12). Furthermore, because the chemical oxidation of cysteine in growth medium produces cystine, our test strain also lacked the TcyJLN and TcyP cystine importers.

**FIG 2:**
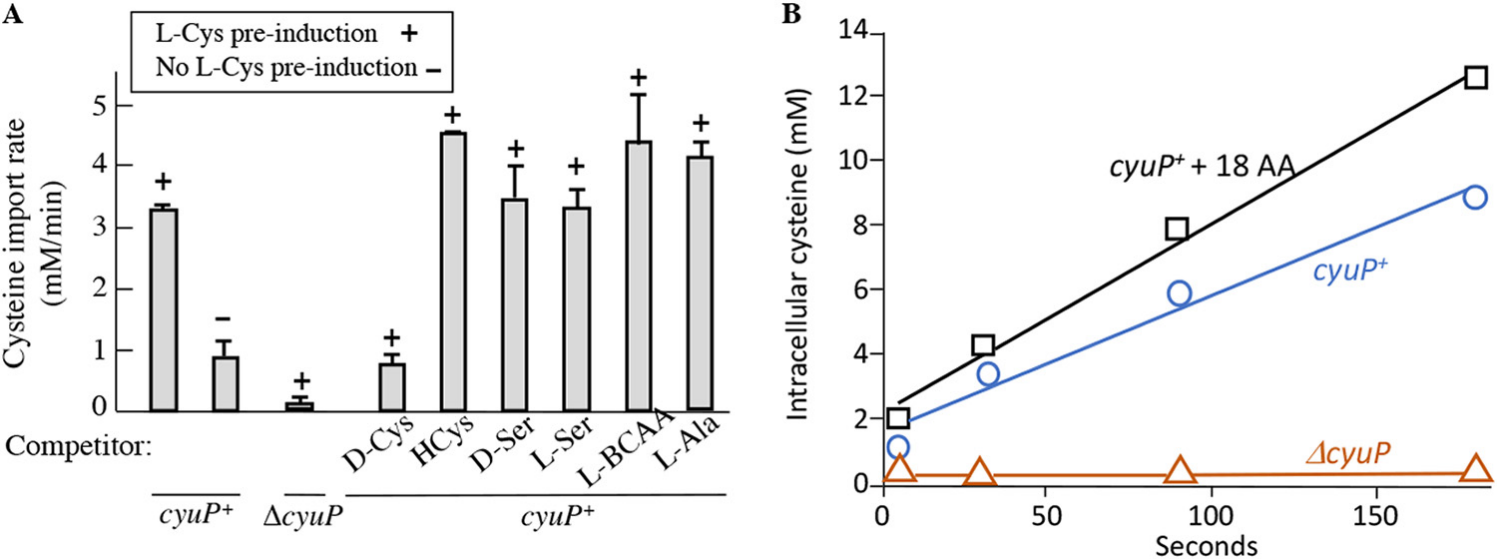
CyuP import activity is specific for cysteine. (A) Cells were precultured in 15 mM pyruvate/ammonium sulfate medium. As noted, 10 mM cysteine was added for two doublings to induce *cyuP*. Cells were harvested, and the import of [^14^C]cysteine (20 μM) was measured. Where indicated, a 10-fold excess of competitors was included in the transport assay. The strains used are ZYD254 and ZYD256. HCys, homocysteine; BCAA, branched-chain amino acid. (B) Example time courses of cysteine import into *cyuP^+^* and Δ*cyuP* cells that had been grown in pyruvate/cysteine medium. The [^14^C]cysteine concentration was 20 μM. In one curve (18 AA), the 18 nonsulfurous amino acids (500 μM each) were added, each in a 25-fold excess to cysteine. The strains used are ZYD254 and ZYD256.

This strain was preinduced with 10 mM cysteine, the cells were washed, and 20 μM radiolabeled cysteine was then provided as the substrate. Substantial cysteine import was detected (Fig. 2). Activity depended entirely upon *cyuP* and was much lower without induction. The results confirmed that CyuP is a cysteine importer and that its induction requires cysteine exposure.

A 10-fold excess of related amino acids did not inhibit cysteine import, showing that the activity of CyuP is specific to cysteine. To date, it is the only dedicated cysteine importer that has been functionally validated among the bacteria.

**FIG 3:**
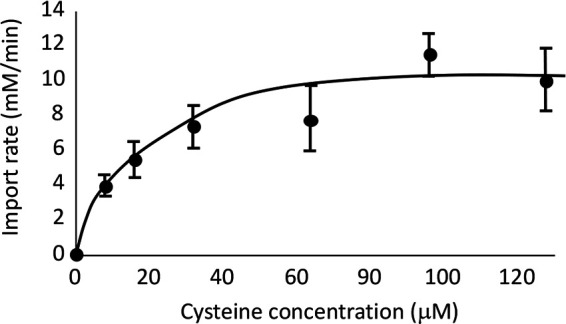
The apparent *K_M_* of CyuP is 20 μM. A strain lacking four nonspecific cysteine importers (Δnull) was cultured in pyruvate/cysteine medium, washed, and tested for cysteine import. Half-maximal transport occurred at approximately 20 μM. The Δ*cyuP* mutant did not exhibit detectable import even at 100 μM cysteine (not shown). The strain used is ZYD254.

The apparent *K_M_* for transport was 20 μM (Fig. 3; Fig. S3). This value is significantly higher than those of importers that have evolved to supply the cell with amino acids that are directly assimilated into proteins; their *K_M_* values are generally <5 μM for ion-driven transporters and <1 μM for ATP-driven transporters (12). However, high (>10 μM) values are often observed for importers that are associated with amino acid catabolism. Examples include the tryptophan importer TnaB (*K_M_* = 70 μM) and the DcuA aspartate importer (*K_M_* = 43 μM) (13, 14). The catabolic transporters can also be differentiated from assimilatory transporters by their regulation, as they are often induced rather than repressed when their substrate is abundant, and by their genomic proximity to genes encoding enzymes that catabolize their substrates. These criteria fit the bicistronic arrangement of *cyuP* and *cyuA*, raising the prospect that this operon serves the purpose of cysteine catabolism. To gather further evidence, we examined the presumptive desulfidase activity of CyuA.

10.1128/mBio.02965-21.4 FIG S3 The apparent *K_M_* of CyuP is 20 μM. Cells were grown in 15 mM pyruvate/ammonium medium containing 10 mM cysteine as an inducer. Import was measured in the presence of 2 mM of Leu, Ile, Val, and Ala (each) to block nonspecific import, primarily by the LIV system (12). The dose-response curve and apparent *K_M_* resemble those of the Δnull strain. The strain used is KCI1266. Download FIG S3, TIF file, 0.8 MB. Copyright © 2022 Zhou and Imlay. 2022 Zhou and Imlay. https://creativecommons.org/licenses/by/4.0/ This content is distributed under the terms of the Creative Commons Attribution 4.0 International license.

### E. coli CyuA is a cysteine-specific desulfidase.

Cysteine desulfidation is analogous to catabolic serine dehydration, with the removal of H_2_S rather than H_2_O: cysteine → pyruvate + NH_4_^+^ + H_2_S (reaction 1) and serine → pyruvate + NH_4_^+^ + H_2_O (reaction 2). Many dehydratases, including some serine dehydratases, use [4Fe-4S] clusters to both bind their substrates and then abstract the hydroxyl leaving groups. Such clusters are solvent exposed, making them vulnerable to both adventitious oxidation and spontaneous iron dissociation (15, 16). CyuA exhibits sequence similarity to these dehydratases, and in fact, workers had reported both that M. jannaschii CyuA (then called MJ1025) has an iron-sulfur cluster and that it loses activity if handled in oxic buffers (3). Therefore, we purified and characterized E. coli CyuA in an anaerobic chamber. The as-isolated enzyme still showed virtually no activity, but activity was recovered when it was incubated with Fe(II) and dithiothreitol (DTT). This treatment can reactivate dehydratases that have damaged iron-sulfur clusters. It is unlikely that CyuA was fully reactivated, so we were unable to determine a reliable *k*_cat_.

**FIG 4:**
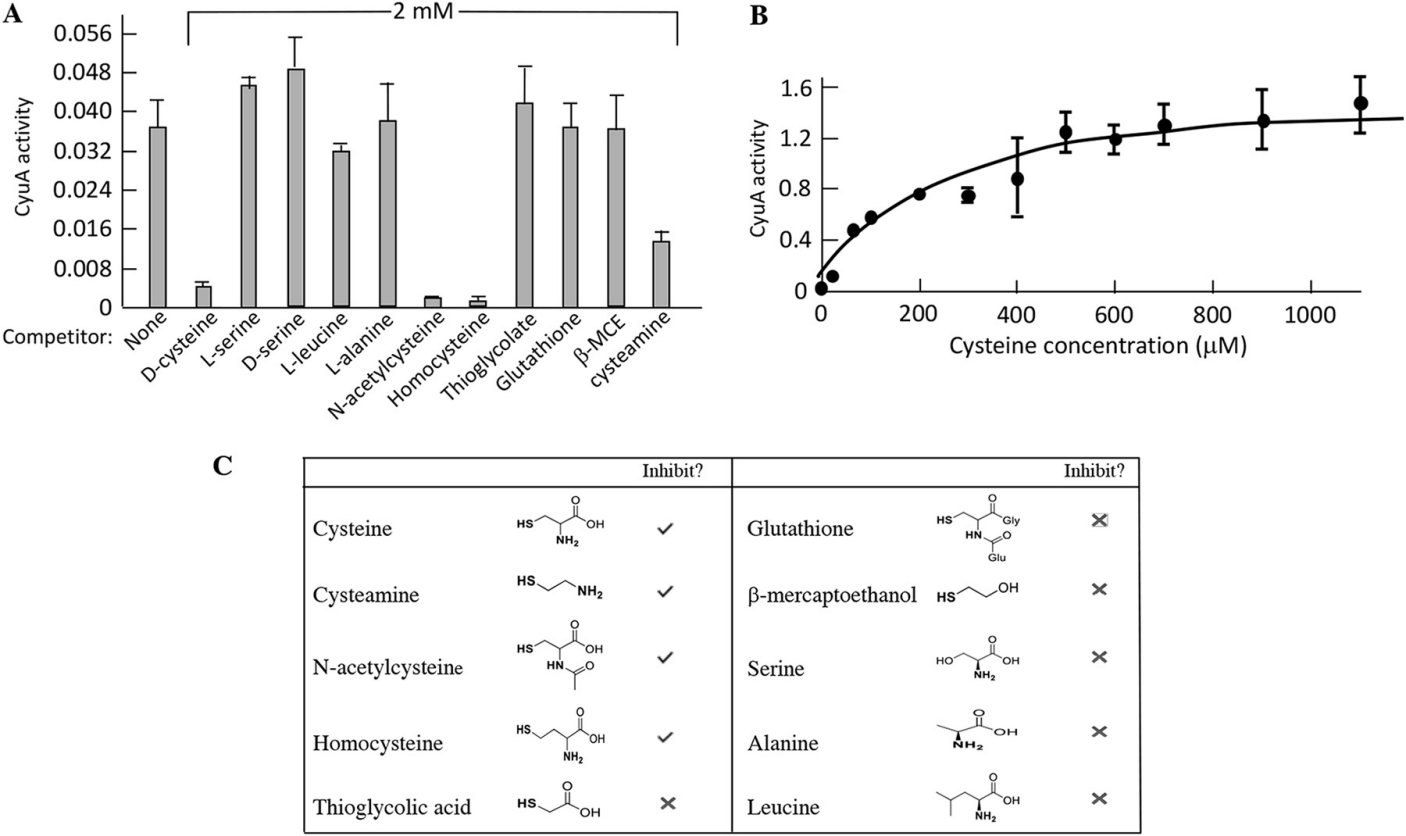
Characterizing the CyuA protein. (A) CyuA was assayed with 200 μM cysteine and 2 mM potential competitors. β-MCE, β-mercaptoethanol. (B) The activity of purified CyuA exhibited a half-maximum rate at 200 μM cysteine. (C) Structures of compounds tested in panel B. CyuA tends to bind compounds that contain a thiol and a primary amine.

The reactivated enzyme exhibited activity with l-cysteine but not with d-cysteine, serine, or alanine (Fig. S4). A few thiol compounds could inhibit the enzyme when they were present in a 20-fold excess, but again, neither serine nor alanine was competitive (Fig. 4A). Therefore, the enzyme is specific for l-cysteine. Notably, the *K_M_* was 200 μM (Fig. 4B). During routine growth in sulfate medium, E. coli maintains an internal cysteine pool of ∼55 μM, which is sufficient to saturate its cysteinyl-tRNA synthetase (*K_M_* = 7 to 25 μM) (17); the higher *K_M_* of CyuA may ensure that it does not deplete the cysteine pool to the point that protein synthesis is impaired.

10.1128/mBio.02965-21.5 FIG S4 Analogs of l-cysteine are not substrates of CyuA. (A) Time course of l-cysteine degradation by CyuA, as indicated by enzyme-coupled NADH oxidation. (B) Conversion to pyruvate was measured using purified CyuA. Two millimolar each amino acid was added. Only l-cysteine gave a positive reaction. Download FIG S4, TIF file, 0.9 MB. Copyright © 2022 Zhou and Imlay. 2022 Zhou and Imlay. https://creativecommons.org/licenses/by/4.0/ This content is distributed under the terms of the Creative Commons Attribution 4.0 International license.

### CyuP and CyuA allow E. coli to use cysteine as a carbon or nitrogen source.

Our main goal was to define the physiological role of CyuPA. Previous workers had not observed growth when cysteine was provided as the sole carbon source. However, when we supplied 10 mM, a very long (40-h) growth lag was followed by successful outgrowth (Fig. S5). When the outgrowers were then isolated and retested, they behaved like the original strain, indicating that they were not suppressors. It seemed plausible that cell metabolism was initially inhibited by the high cysteine concentration that we had abruptly imposed, creating a crisis in which CyuP and CyuA could not promptly be induced. Indeed, we found that the lag could be eliminated by preculturing cells with pyruvate and 1 mM cysteine to enable the induction of both CyuPA and pyruvate dissimilatory enzymes. We note that cysteine concentrations in natural environments are likely to be micromolar, not millimolar (18–20).

10.1128/mBio.02965-21.6 FIG S5 A long lag (>1 day) can occur when cysteine abruptly replaces a prior carbon source. Cells were precultured in anoxic medium containing ammonium and 22 mM pyruvate. They were then washed and inoculated into anoxic medium containing only 10 mM cysteine as the carbon source. The bacteriostatic effect of abundant cysteine likely delays the induction of CyuA. The strain used is ZYD156. Download FIG S5, TIF file, 1.1 MB. Copyright © 2022 Zhou and Imlay. 2022 Zhou and Imlay. https://creativecommons.org/licenses/by/4.0/ This content is distributed under the terms of the Creative Commons Attribution 4.0 International license.

**FIG 5:**
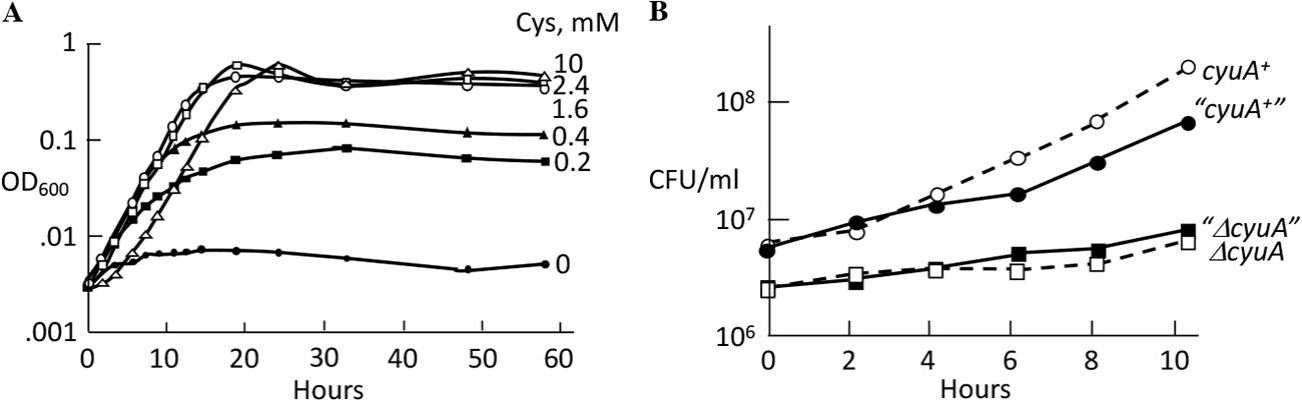
Cysteine can be used as the sole nitrogen source. (A) Cells were precultured in glucose-glutamine medium and then washed and incubated in glucose medium containing the indicated amounts of cysteine as the sole nitrogen source. The strain used is KCI1266. (B) Cysteine (1 mM) utilization as nitrogen relies mainly on CyuA. Cultures contained glucose with cysteine as the nitrogen source. Strains in the mixed culture are indicated in quotation marks, while parallel pure cultures have standard labels. The produced ammonia was predominantly trapped within the cell and assimilated as a nitrogen source, rather than being released out into the environment, as the CyuA^+^ strain did not cross-feed a *cyuA* mutant. The strains used are KCI1266 and SSK195. This and other time courses are representative of multiple replicates.

Cells were able to grow using cysteine as their sole nitrogen source by capturing the ammonium ion that is released by CyuA (Fig. 5A). Approximately 1.6 mM cysteine enabled cells to reach the same density as with 0.8 mM glutamine (not shown), which is in accord with the fact that cysteine provides a single nitrogen atom, while glutamine provides two. Mutants lacking *cyuA* could not grow. In a mixed-culture experiment, the CyuA-proficient strain did not cross-feed a CyuA-deficient mutant, showing that most or all of the ammonium released by CyuA was trapped by glutamine synthetase before it could escape the cell (Fig. 5B). This is impressive because the predicted escape time is ∼15 ms (see the supplemental material). Thus, cysteine degradation is an efficient mechanism of nitrogen acquisition.

**FIG 6:**
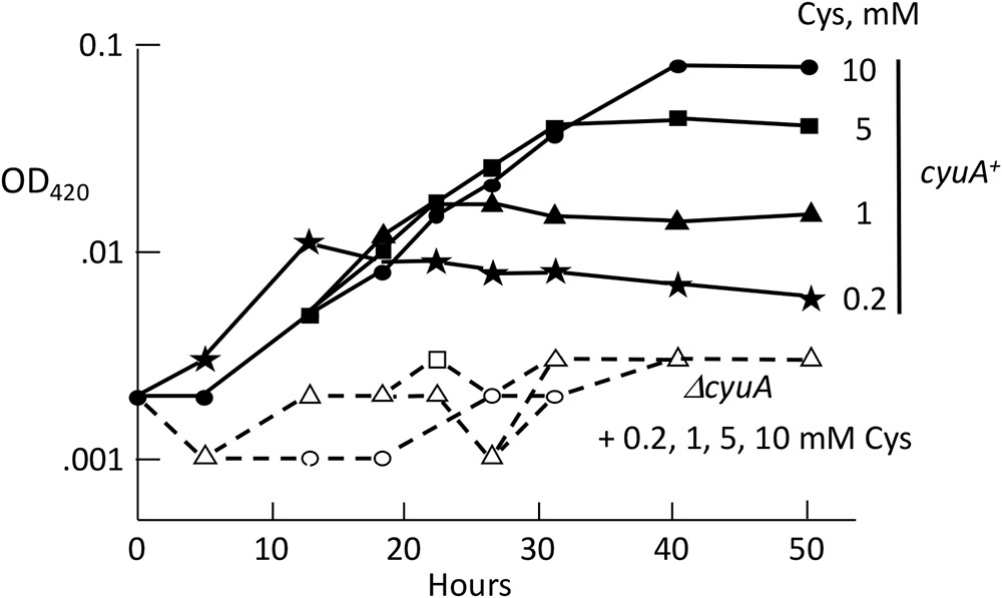
Cysteine can be used as the sole carbon source. Cells were precultured in anaerobic pyruvate-ammonium medium, washed, and suspended in cysteine-ammonium medium in which the indicated amounts of cysteine were the sole carbon sources. Growth depended upon CyuA. The OD was monitored at 420 nm to improve sensitivity at low cell densities. The strains used are KCI1266 and SSK195.

**FIG 7:**
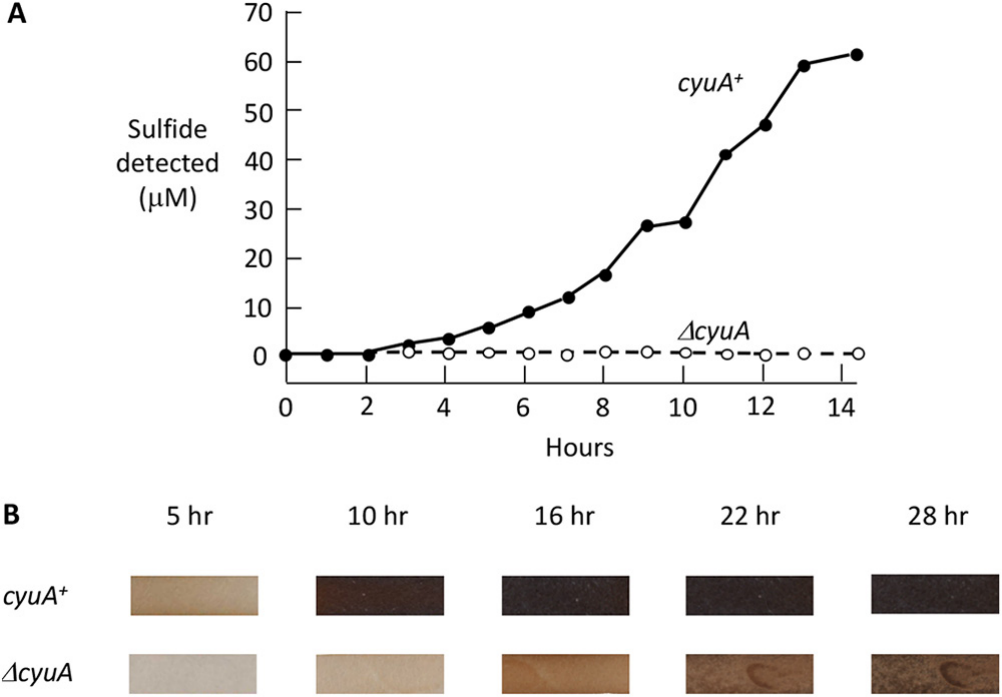
CyuA is the primary cysteine-degrading enzyme. (A) Sulfide production with cysteine relies on CyuA. Glucose medium contained cysteine (1 mM) as the sole nitrogen source. Sulfide detection is relative, as some is lost into the gas phase. The strains used are KCI1266 and SSK195. (B) Cells were top spread onto pyruvate-ammonium plates, and sterile discs soaked in 1 M cysteine were placed in the middle. Lead acetate strips were taped onto the lids and photographed at intervals. Mutants lacking *cyuA* released sulfide more slowly than did the parent strain. The residual sulfide release from the Δ*cyuA* mutant likely arises from the adventitious cysteine desulfurase activities of other enzymes (see the text). The strains used are KCI1266 and SSK195.

Similarly, cysteine was able to support growth by serving as the sole carbon source (Fig. 6). Again, CyuA was required. Parallel experiments showed that CyuA was the predominant catalyst of cysteine depletion from the medium, while lead acetate strips indicated that it was the primary source of sulfide production (Fig. 7). A slower release of sulfide was observed with *cyuA* mutants, probably reflecting the adventitious desulfidation activities of tryptophanase (TnaA), 3-mercaptopyruvate sulfurtransferase (MstA or SseA), *O*-acetylserine sulfhydrylases, or cystathionine β-lyases (21–23). Such nonspecific activities are highest when cysteine concentrations are elevated, as they will be when a *cyuA* mutant is incubated with high-millimolar cysteine.

The quantitative data indicated that the changes in biomass fit the measured rates of cysteine degradation (see the supplemental material); thus, all the pyruvate and ammonium were consumed. The data also predicted that when cysteine was used as a nitrogen source, with a doubling time of 144 min, the required rate of cysteine import would be 27 mM/min, normalized to the intracellular volume. When used as a carbon source (Doubling time, *t_D_* = 350 min), the import rate would be 170 mM/min. These are strikingly high rates; in comparison, the rates of cysteine assimilation required for it to serve as the sole sulfur source would be only 0.6 mM/min and 0.25 mM/min at a *t_D_* of 144 min and a *t_D_* of 350 min, respectively (2). The high cysteine demand explains why strong CyuP induction must precede cysteine-dependent growth.

Both the nitrogen demand and carbon demand rates are substantially higher than the 10 to 20 mM/min apparent transport rates that we typically measured in our assays (Fig. 2; Fig. S3). However, during those experiments, most of the imported cysteine was continuously degraded to formate and acetate, which effused back out of the cell; thus, the rate of radiolabel accumulation did not represent the total rate of import.

These results demonstrated that CyuA is essential for cysteine catabolism. In contrast, growth still occurred in CyuP-deficient strains. Our previous work showed that cysteine can enter cells as a pseudosubstrate of importers whose proper substrates are other amino acids (12); their affinity for cysteine is low, but at the millimolar concentrations that are employed in laboratory experiments, the flux is evidently sufficient to enable growth. To more carefully test whether CyuP assists in growth, we conducted mixed-culture experiments in which wild-type and CyuP-deficient strains were competed. Cysteine was provided at 0.2 mM to test its use as a nitrogen source and at 1 mM to test it as a carbon source. These concentrations are sufficient to grow cultures to optical density at 600 nm (OD_600_) values of approximately 0.2 and 0.03, respectively, at which point the cysteine would be exhausted. Therefore, cultures were grown to these points and then repeatedly subcultured. The expectation was that if CyuP assists in cysteine acquisition, especially at the low cysteine concentrations that precede its exhaustion, then the wild-type strain will progressively take over the mixed culture.

**FIG 8:**
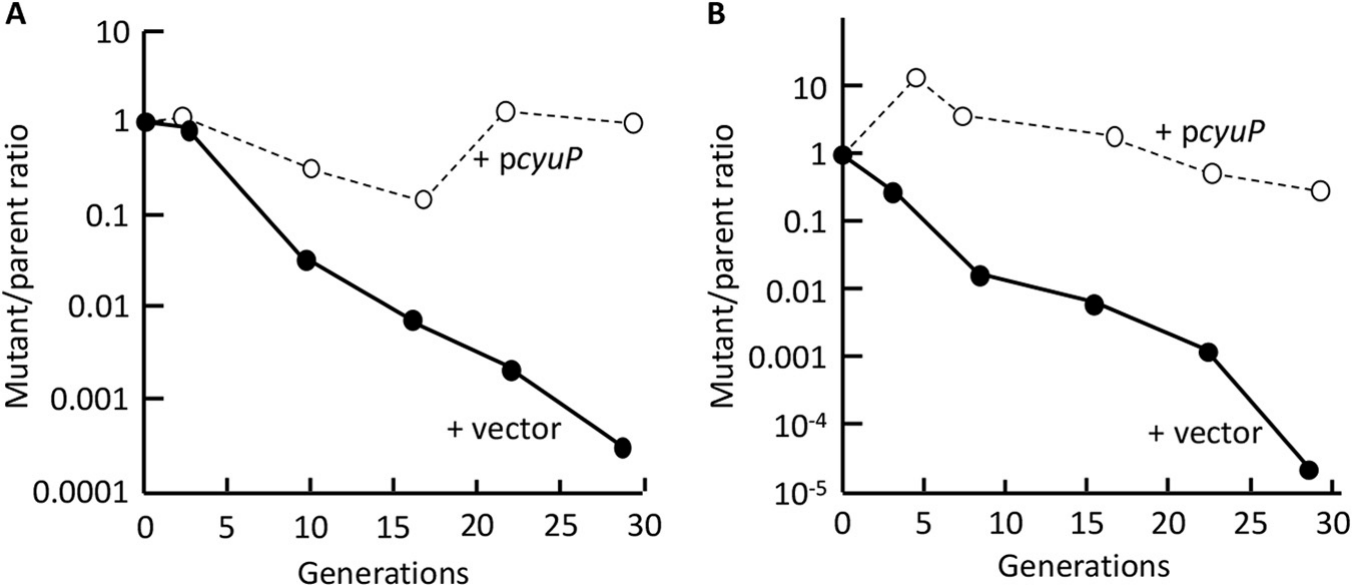
CyuP enhances cysteine utilization. CyuP^+^ strains outcompeted CyuP^−^ mutants when cysteine was provided as the sole nitrogen (A) or carbon (B) source. ZYD286 (Δ*cyuP*, with a vector) was mixed with ZYD290 (*cyuP^+^*, with a vector), and the ratio of the mutant/wild type is graphed (filled circles) over the course of 30 generations, with periodic subcultures (see Materials and Methods). Cysteine was provided at 0.2 mM for use as a nitrogen source with glucose (A) or 1 mM for use as a carbon source with ammonium (B). As a control, the same strains were complemented with a CyuP-expressing plasmid (ZYD288 and ZYD292) (open circles), and the growth disadvantage of the mutant strain was abolished.

This outcome was observed. Figure 8 shows that the wild-type strain outcompeted the transport mutant by multiple orders of magnitude, confirming the value of CyuP in cysteine catabolism.

Those experiments employed a nonpolar deletion of *cyuP* in order to avoid any impact on the expression of the downstream *cyuA* gene. As an additional precaution, we also performed the experiments with a *cyuP*-complementing plasmid. The complemented mutant was not outcompeted.

### The *cyuPA* operon is poorly expressed in a *crp* mutant.

**FIG 9:**
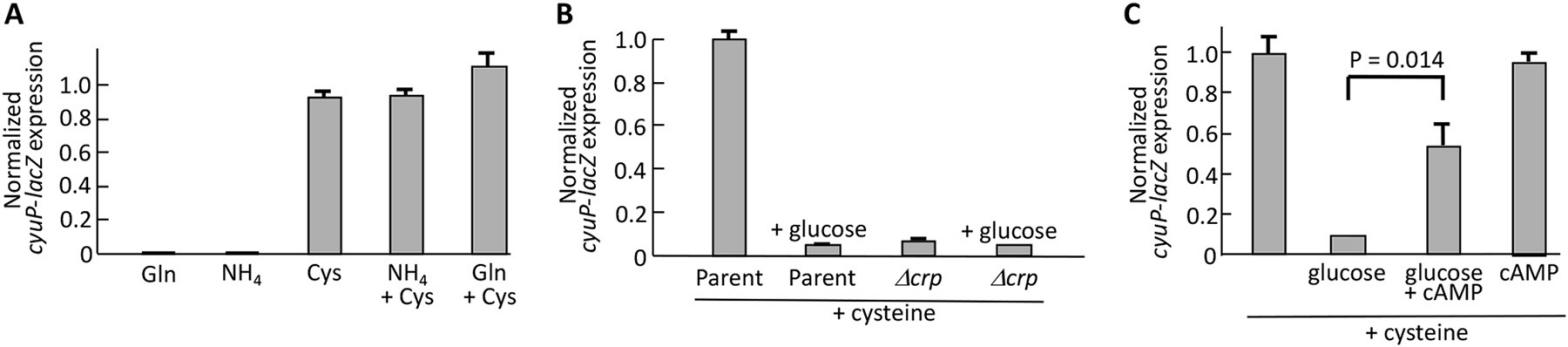
*cyuP* is regulated by Crp and CyuR. (A) Cells were grown for 4 generations in minimal medium with glucose as the carbon source and with glutamine (7.5 mM), cysteine (5 mM), and/or ammonium (15 mM) as the nitrogen source. Cultures were harvested at an OD_600_ of 0.1. The expression of *cyuP-lacZ* depended on cysteine but was unaffected by the identity of the nitrogen source. Data are normalized to the glucose/cysteine sample. The strain used is ZYD156. (B) *cyuP* is positively regulated by both CyuR and Crp. Cells were cultured for 4 generations in LB medium plus 10 mM cysteine before fusion expression was measured at an OD_600_ of 0.2. Glucose (0.2%) repressed expression. The strains used are ZYD156 and ZYD266. (C) Where indicated, 5 mM cAMP was added to LB plus 10 mM cysteine medium to activate Crp. In panels B and C, data are normalized to the LB plus cysteine sample.

E. coli prefers ammonia to glutamine as a nitrogen source: the Nitrogen Regulator I (NRI) and Ntr systems are activated if glutamine is the sole nitrogen source, whereas their transcription is diminished if ammonia is supplied (24, 25). In glucose/cysteine cultures, the expression of the *cyuP-lacZ* fusion was unaffected by whether glutamine or ammonia was provided (Fig. 9A). We infer that nitrogen levels do not control *cyuPA* transcription.

In contrast, the addition of glucose to cysteine cultures did diminish expression (Fig. S6). This result fits a previous observation made in Salmonella (7). We suspected that the catabolite repressor protein Crp might be involved, as this transcription factor activates the expression of other amino acid catabolic operons (26–29). Because *crp* mutants grow poorly in defined media with weak carbon sources, its involvement was examined in Luria broth (LB) medium (Fig. 9B). In that medium, glucose strongly repressed *cyuP-lacZ* expression despite the presence of cysteine; the addition of cAMP restored strong expression (Fig. 9C). Furthermore, *crp* mutants exhibited very low expression levels even in glucose-free medium. We note that glucose blocked *cyuP* induction more completely in LB than in defined medium. In minimal medium, the inducer cysteine can enter cells through branched-chain transporters (12) and does not require CyuP induction; in contrast, in rich medium, those transporters are occupied by their cognate substrates, and cysteine entry requires CyuP. The latter arrangement creates a feedforward expression system in which cysteine induces its own uptake. Feedforward systems amplify the impact of any regulator of the operon, including, in this case, Crp.

10.1128/mBio.02965-21.7 FIG S6 Glucose suppresses the cysteine import rate, but ammonium does not. Cells were grown from an OD of 0.003 to 0.012 in three media: 22 mM pyruvate–15 mM NH_4_, 0.2% glucose–15 mM NH_4_, and 0.2% glucose–1 mM glutamine. After the OD reached 0.012, 10 mM cysteine was added. Cells were harvested at an OD of 0.1 and resuspended in glucose-ammonium medium for transport assays. ^14^C-20 μM cysteine transport rates (within 45 s) were measured. The strain used is ZYD254. Download FIG S6, TIF file, 1.2 MB. Copyright © 2022 Zhou and Imlay. 2022 Zhou and Imlay. https://creativecommons.org/licenses/by/4.0/ This content is distributed under the terms of the Creative Commons Attribution 4.0 International license.

**FIG 10:**
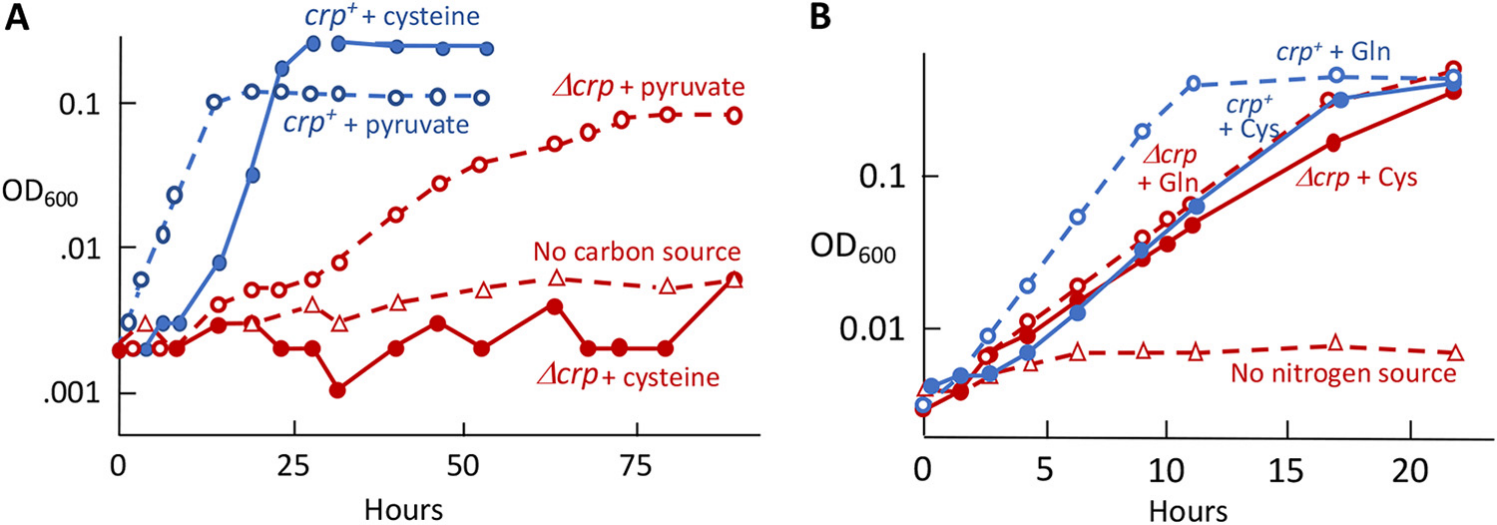
Cysteine cannot serve as the sole carbon source in Δ*crp* mutants. (A) Cells were precultured in medium containing 22 mM pyruvate and 1 mM cysteine. They were then washed and resuspended in medium that contained either 20 mM cysteine or 20 mM pyruvate as the sole carbon source. The Δ*crp* mutants were able to catabolize pyruvate but not cysteine. The strains used are ZYD156 and ZYD266. (B) Cysteine suffices as a nitrogen source even in Δ*crp* mutants. Cells were precultured in glucose medium containing 1 mM glutamine and 1 mM cysteine. They were washed with nitrogen-free medium and resuspended in medium containing either 10 mM cysteine or 5 mM glutamine as the sole nitrogen source. The strains used are ZYD156 and ZYD266.

The impact of Crp on cysteine catabolism was also evident in growth curves. Null *crp* mutants were unable to grow when cysteine was provided as the sole carbon source (Fig. 10A). Crp assists in the control of pyruvate dissimilation, but this was not the full explanation because the strains retained the ability to ferment pyruvate.

In contrast, *crp* was not necessary for cells to use cysteine as a nitrogen source (Fig. 10B). The reduced level of *cyuPA* expression in glucose medium is evidently still sufficient for the lower cysteine flux needed as a nitrogen source. More work will need to be done to establish the details of Crp control, including the question of whether Crp acts directly at the *cyuPA* locus. We generated a *cyuR-lacZ* fusion but did not see any control of its expression in LB medium by glucose or Crp. In any case, the involvement of Crp in *cyuPA* expression and function fits the model that the primary purpose of this operon is to exploit cysteine as an energy source.

### CyuA is not functional in an oxic environment.

**FIG 11:**
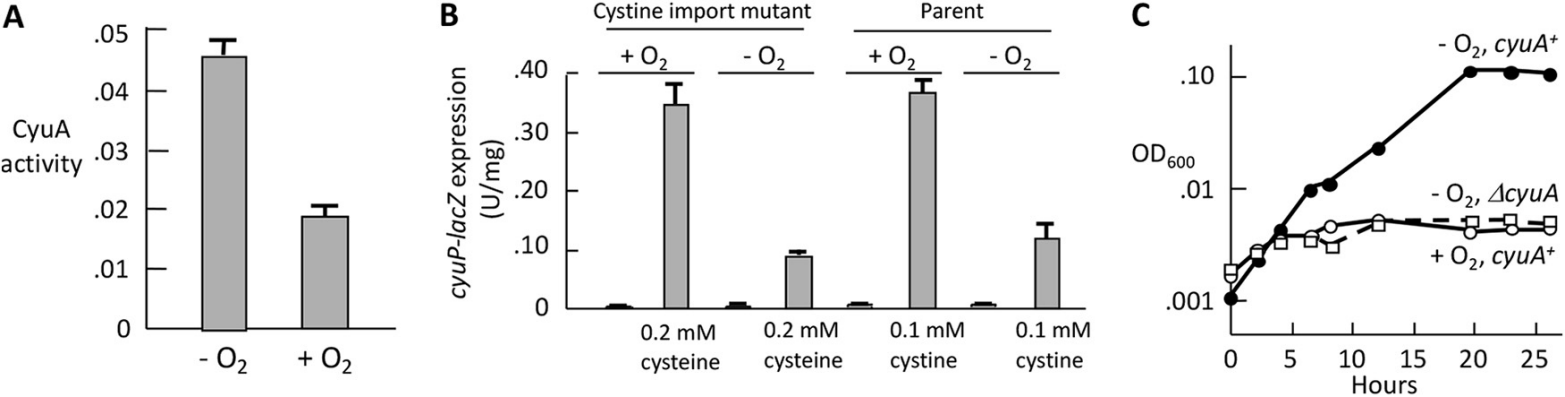
CyuA is sensitive to oxygen. (A) Purified CyuA is inactivated by oxygen exposure. CyuA was assayed with cysteine in a sealed anaerobic cuvette. SOD (60 U/mL) and catalase (60 U/mL) were added to prevent the accumulation of H_2_O_2_ and superoxide, and 100 μM DTPA was added to prevent any possible Fenton reaction. After an anoxic assay for 2 min, the cuvette lid was opened, and air was introduced by pipetting the reaction mixture down the wall of the cuvette. Bars represent reaction rates during the 2 min before and after aeration. Reaction rates remained steady under anoxic conditions (not shown). (B) CyuP is induced under both aerobic and anaerobic conditions. Cells were grown in glucose-ammonium medium under oxic or anoxic conditions, as indicated. At an OD of 0.1, either 0.2 mM cysteine (ZYD156, lacking cystine importers) or 0.1 mM cystine (ZYD277) was added; β-galactosidase from *cyuP-lacZ* was measured at an OD of 0.2. DTNB measurements showed that 70% of the cysteine was still in the medium at the point of harvest, confirming that cysteine had not been depleted by oxidation. The strains used are ZYD156 and ZYD277. (C) Cystine can be used as a nitrogen source by CyuA under anaerobic but not aerobic conditions. At time zero, cells precultured in glucose-ammonia medium were washed and suspended in glucose medium containing only cystine as a nitrogen source. Strains that express a cystine importer (TcyP) in the presence of tetracycline (8 μg/mL) were used. Imported cystine is reduced to cysteine intracellularly. The strains used are SSK167 and ZYD285.

When we overproduced CyuA in anoxic cultures but lysed cells in the open laboratory, no activity was recovered, even when subsequent purification steps were performed inside an anaerobic chamber. Activity could be regained upon Fe(II)/DTT treatment, suggesting that the CyuA catalytic [4Fe-4S] cluster might be unusually unstable to brief oxygen exposure. This idea was directly tested. The purified reactivated enzyme was added to a sealed anaerobic cuvette along with other assay components, including cysteine. The reaction was tracked for 2 min. The sealed lid was then opened, and the reaction mixture was aerated by pipetting the solution down the walls of the cuvette. The reaction rate was then remeasured and determined to be reduced by one-half (Fig. 11A). Because catalase and superoxide dismutase (SOD) were included in the reaction mix, we deduce that the active oxidant was molecular oxygen itself. We have not previously observed this degree of oxygen sensitivity for other enzymes of the dehydratase class (30, 31). More work will need to be done to quantify and explain this effect.

Given those results, we wished to test whether CyuA is functional inside aerobic cells. It is tricky to do so using cysteine in the growth medium, as cysteine is quickly oxidized. An alternative was to supply cystine, which is rapidly reduced to cysteine when it enters the cell (1, 11). Our previous transcriptome sequencing (RNA-seq) data showed that *cyuPA* is strongly induced when cystine is added to an aerobic culture (1), and we found that the *cyuA-lacZ* fusion is actually more strongly expressed under oxic than anoxic conditions (Fig. 11B). However, the burgeoning intracellular pool of cysteine leads to the immediate deactivation of CysB (32), and the transcription of the cystine importers is turned off (1), which would preclude the use of cystine as a high-flux carbon or nitrogen source. (Indeed, wild-type cells did not grow when cystine was supplied as the sole carbon source.) To circumvent this problem, we expressed the cystine importer TcyP from a tetracycline-driven promoter so that sufficient cystine influx continued throughout the growth period. The CyuA^+^ cells were able to use cystine as a nitrogen source under anoxic conditions, but they were unable to do so under oxic conditions (Fig. 11C). It is odd that E. coli is capable of transcribing the *cyuPA* operon under oxic conditions when its CyuA gene product is not functional (see Discussion).

## DISCUSSION

E. coli possesses transporters for all of the proteogenic amino acids, indicating that they are at least occasionally available in its natural habitat. However, the bacterium uses relatively few of them as catabolic substrates, including several—Asp, Asn, Pro, and Ala—that can be consumed only when an external electron acceptor is available (33). Cysteine now joins only serine, threonine, and possibly tryptophan as amino acids that E. coli can ferment.

**FIG 12:**
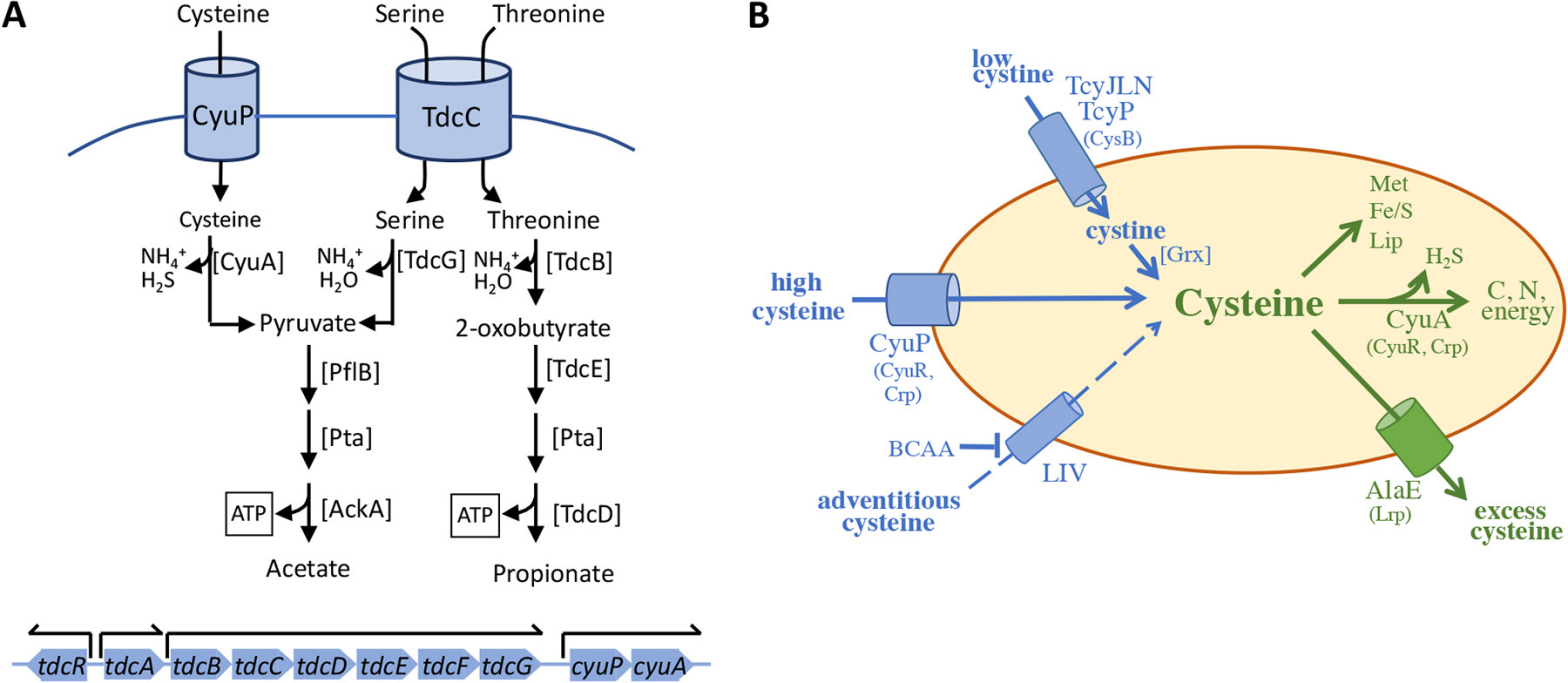
The place of cysteine degradation in metabolism. (A) Anaerobic catabolism of cysteine, serine, and threonine. All three pathways start with deaminating dehydration reactions. CyuA, PflB, and TdcE are oxygen-sensitive enzymes. TdcR and TdcA regulate the *tdc* operon; TdcF is an enamine/imine deaminase that may assist TdcB. At the bottom, the *cyu* and *tdc* operons are adjacent but independently transcribed. (B) Overall scheme of cysteine flow in E. coli. Trace cystine is imported by TcyP and TcyJLN, and it is then reduced to cysteine. Cysteine itself can be adventitiously imported by transporters such as the LIV system, but this uptake is blocked when their native amino acid substrates are present. No system has yet been found that is dedicated to scavenging trace environmental cysteine. When it is abundant, cysteine can be imported by CyuP and, under anoxic conditions, degraded by CyuA for use as a carbon or nitrogen source. Excess cytoplasmic cysteine from uncontrolled cystine import is exported by AlaE. The transcriptional regulators that control these processes are indicated in parentheses.

Other bacteria employ specialized pathways dedicated to the fermentations of alanine, lysine, arginine, glutamate, and other amino acids, and Stickland-type pathways enable the cofermentations of even more (34). In contrast, E. coli primarily constrains its fermentations to those amino acids that can be converted to pyruvate in a single step. From there, the downstream pathway uses the same enzymatic machinery that ferments carbohydrates. Threonine catabolism is a modest exception (Fig. 12A).

It is not known whether amino acid catabolism contributes to E. coli growth in the gut. Analyses of total colonic contents show that some amino acids are present at low-micromolar concentrations (18, 19). These levels will probably vary upon the proximity to the duodenum, whereby dietary amino acids might enter, and the epithelium, which might be a source of degradable proteins. In the latter case, bacteria like E. coli that reside near the epithelial surface might encounter a higher amino acid concentration than is suggested by the total-colon values. Other amino acids may be released within the lumen from the death and degradation of local microbes. Strikingly, the reported colonic cysteine levels are vanishingly small—low nanomolar at best—either because cysteine is absent or, perhaps, because the harvesting protocol failed to protect cysteine from oxidation (20). The upshot is that those studies do not provide insight on where CyuP/CyuA might benefit E. coli.

The CyuP/CyuA system possesses characteristic traits of catabolic pathways: a polycistronic operon that includes both transporter and degrading enzymes, Crp-dependent transcription, induction by the substrate, a high flux capacity, and a relatively high *K_M_* for transport and catabolism. The full process would directly yield 1 ATP per cysteine catabolized, minus the equivalent of 1/3 ATP expended for import. In comparison, E. coli glucose fermentation yields 3 net ATP. These yields are roughly proportional to growth rates, as the doubling time on cysteine was 350 min, and that on glucose was 60 min. Cysteine is among the least energetic substrates that E. coli ferments, which explains why a high flux is necessary to support even a relatively low growth rate. Serine and threonine would match cysteine in energy yield (Fig. 12A). Notably, threonine and serine catabolism are encoded by genes that sit in a common operon (*tdcABCDEFG*), and this operon lies directly upstream of *cyuPA*. The two operons have discrete promoters (5); cysteine accumulation induces *cyuPA* without any impact on *tdc* (1). However, the gene arrangement suggests that the *tdc-cyu* region might move through the biota as a locus that allows amino acid fermentation.

### Cysteine-catabolic regulons vary in their gene content.

It is puzzling, and intriguing, that various *Enterobacteriaceae* contain distinct combinations of regulator, transporter, and desulfidase. The CyuR regulator is present in all strains. Nonaka and Takumi found that some species possess only the CyuA desulfidase, some have CdsH, and some have both (35). The CyuP importer is usually present in those bacteria that use CyuA, but CdsH-only strains lacked CyuP, raising the question of how the cysteine might enter. Cysteine can be adventitiously imported by transporters whose true substrates are branched-chain amino acids, with which cysteine shares a nonpolar side chain (12). In the study reported here, nonspecific cysteine import still enabled substantial cysteine-dependent growth of *cyuP* mutants albeit not as much as when CyuP was present.

Why do some bacteria like Salmonella have two CyuR-controlled desulfidases? The obvious functional difference between the two enzymes is the oxygen sensitivity of CyuA, and indeed, Salmonella reciprocally regulates the desulfidases so that CyuA is expressed in anoxic environments and CdsH is optimally expressed in oxic ones (6). We speculate that when Salmonella enters aerated habitats, the oxygen tolerance of pyridoxal 5′-phosphate (PLP)-dependent CdsH allows the catabolism of cysteine to continue. The converse question is why CdsH would not also suffice under anoxic conditions, and the answer is not clear. The CdsH turnover number is high (36, 37), making it unlikely that CyuA is preferred for kinetic reasons. However, Kredich et al. noted that sulfide can inhibit CdsH (*K_I_* = 10 μM) (36). In aerobic habitats, CdsH may avoid poisoning itself because the hydrogen sulfide that it generates will rapidly diffuse out of the cell and be lost to the world. However, when growing in the anoxic gut, enteric bacteria are surrounded by sulfur-reducing bacteria that generate up to millimolar levels of environmental sulfide (38). We speculate that this sulfide will permeate all residents and may be enough to block CdsH turnover.

### The oxygen sensitivity of CyuA is biochemically unexpected.

Although CyuA is a desulfidase, its reaction mechanism mirrors that of [4Fe-4S]-dependent dehydratases that remove H_2_O, rather than H_2_S, from their substrates. The cluster serves as a Lewis acid that both binds the substrate and abstracts OH^−^ (or HS^−^) from it. Such clusters are destabilized when molecular oxygen oxidizes them from their +2 to +3 valence; the catalytic Fe(II) then dissociates, leaving behind a [3Fe-4S]^+^ partial cluster that is catalytically inactive. Dehydratases typically exhibit a 20- to 30-min half-life when exposed to full aeration; because cells continuously repair them, they retain enough activity to be highly active even inside aerobic cells, where they play key roles in central metabolism and biosynthetic pathways (30). However, in our experiments, purified CyuA exhibited an aerobic half-life of only ∼2 min, and it failed to degrade cysteine in aerobic cells. We conjecture either that the CyuA cluster is unusually solvent exposed or that it has an especially low reduction potential. This needs to be tested. It is not immediately obvious how such features would optimize desulfidation.

### Are there other cysteine importers?

At this point, CyuP is currently the only proven, dedicated cysteine importer in the microbial world (Fig. 12B). Loddeke et al. determined that CyuP is found only in facultative and anaerobic bacteria, presumably because in oxic environments, cysteine is quickly oxidized to cystine (6). As one might expect, aerobes (as well as anaerobes) possess importers of cystine (2). However, CyuP is a high-flux system that serves a catabolic purpose; it is not a typical high-affinity amino acid importer that supports protein synthesis (12). Workers are still looking for a cysteine importer that does so. Recently, Sabrialabe et al. suggested that the TcyJLN ATP-dependent cystine importer, which is found in E. coli and numerous other bacteria, might do double duty as an importer of cysteine too. This suggestion derived from the ability of the periplasmic TcyJ protein to bind cysteine *in vitro* (39). The idea is intriguing—CysB control of *tcyJLN* would optimize cysteine import when the cell is sulfur poor—but the binding constant (15 μM) is somewhat high, and initial physiological experiments did not detect evidence of its action (12). Furthermore, this transporter is found in committed aerobes too, which, based on the distribution of CyuP, may not normally encounter cysteine.

Another periplasmic binding protein with a much higher affinity for cysteine (CjaA [*K_d_* {dissociation constant} = 0.5 μM]) was isolated from Campylobacter jejuni, but it has not yet been functionally tested (40). That transport system is sparingly scattered among bacteria and is absent from *Enterobacteriaceae*. We have conjectured that the toxicity of cytoplasmic cysteine may have suppressed the evolution of cysteine importers, other than those that are partnered with degrading enzymes (12). Finally, Legionella pneumophila is an intracellular pathogen that requires cysteine import for growth: it can neither synthesize its own cysteine nor import cystine (41). Its unique environment ensures that it has access to (reduced) cysteine. High-affinity cysteine uptake by L. pneumophila has been detected in laboratory experiments, but L. pneumophila lacks CyuP, TcyJLN, and any homolog of the high-affinity CjaA importer. Thus, the new findings about CyuP comprise an advance in our understanding of how bacteria use environmental cysteine, but cysteine still stands as the one amino acid whose transport is incompletely understood.

## MATERIALS AND METHODS

### Chemicals.

l-Cysteine hydrochloride monohydrate, l-cystine dihydrochloride, d-cysteine, and other nonradioactive amino acids were purchased from Sigma-Aldrich. Glutathione, homocysteine, cysteamine, thioglycolic acid, *N*-acetylcysteine, sodium pyruvate, NADH, ferrous ammonium sulfate, superoxide dismutase from bovine erythrocytes, l-lactic dehydrogenase from rabbit muscle, ferric chloride, imidazole, l-(+)-arabinose, isopropyl β-d-1-thiogalactopyranoside (IPTG), 5,5′-dithiobis(2-nitrobenzoic acid) (DTNB), *N*,*N*-diethyl-*p*-phenylenediamine (DPD), sodium sulfide, a 30% (wt/wt) solution of hydrogen peroxide (H_2_O_2_), bovine catalase, ovalbumin, *o*-nitrophenol-β-galactoside (ONPG), diethylenetriamine pentaacetic acid (DTPA), dithiothreitol (DTT), β-mercaptoethanol, and antibiotics were purchased from Sigma-Aldrich. Coomassie reagent was obtained from Thermo Scientific. Blank paper discs (6-mm diameter) were purchased from Becton, Dickinson and Company. Medium reagents and buffer chemicals were obtained from Fisher Chemical. Restriction and ligation enzymes were obtained from New England BioLabs. Laemmli sample buffer (2×) was purchased from Bio-Rad. Qiagen kits were used for genomic and plasmid DNA preparation. Whatman lead acetate papers, colony PCR beads, and His GraviTrap were purchased from GE Healthcare. PCR reagents were purchased from Invitrogen. DNA sequencing was performed at ACGT, Inc. Radiolabeled l-[1,2,1′,2′-^14^C]cystine was purchased from Perkin-Elmer. Transport measurement filters (catalog number GSWP02500) and Amicon Ultra centrifugal filters (10,000 molecular weight [10K]) were purchased from Millipore.

### Growth media.

Anaerobic glucose-ammonium growth medium was comprised of minimal A salts (42) supplemented with 0.2% glucose, 0.02% MgSO_4_, and 0.005 mg/mL thiamine. In general, minimal A salts contain K_2_HPO_4_, KH_2_PO_4_, (NH_4_)_2_SO_4_, and sodium citrate · 2H_2_O, adjusted to pH 7.0. For nitrogen-free (glucose-containing) medium, the (NH_4_)_2_SO_4_ from minimal A salts was replaced with KCl of the same molarity (0.15 M). For carbon-free (ammonium-containing) medium, glucose was omitted. Media were stored in a Coy anaerobic chamber under an atmosphere of 90% nitrogen, 5% hydrogen, and 5% carbon dioxide. Histidine (0.5 mM) was included in all anaerobic growth media because wild-type MG1655 exhibits anaerobic bradytrophy. For aerobic growth, histidine was excluded because it is unnecessary.

Luria broth (LB) (10 g Bacto tryptone, 5 g yeast extract, and 10 g NaCl per L) (42) was used for strain constructions and some β-galactosidase enzyme assays.

### Strain construction.

Null mutations in *cyuA* or *crp* were generated by the phage λ Red recombination method (43). The targeted open reading frame (ORF) was replaced by the chloramphenicol cassette from pKD3 or by the kanamycin cassette from pKD4, with FRT sites flanking the drug marker on both sides. The FRT-flanked antibiotic resistance markers were eliminated by the FLP activity of pCP20, and the strains were then cured of pCP20 by incubation at a nonpermissive temperature (42°C). This procedure generates nonpolar mutations. Other mutations were transferred to working strains by P1 transduction (42) from mutants that were obtained from the E. coli Genetic Stock Center. All mutations were confirmed by PCR analysis.

A single-copy *cyuP-lacZ* transcriptional fusion (44) was created by amplifying the promoter region, consisting of approximately 500 bp upstream of *cyuP* through its ATG (+1) site. This target region was then ligated to the *lacZ* coding region in pSJ501. The resultant *cyuP-lacZ* fusion was integrated into the genome at the λ attachment site with the help of pSJ130, which includes the lambda integration genes. The final construct was confirmed by PCR and DNA sequencing. Plasmids were purified by a Qiagen kit.

All mutants and plasmids used in this paper are presented in Table 1. LacZ transcriptional fusion primers, deletion primers and cloning primers are presented in Table 2.

**TABLE 1:** Bacterial strains and plasmids

Strain or plasmid	Genotype and/or characteristic(s)^*a*^	Reference and/or source
Strains		
MG1655	F^−^ wild-type E. coli	53
BW25113	*lacI*^q^ *rrnB*_T14_ Δ*lacZ*_WJ16_ *hsdR514* Δ*araBAD*_AH33_ Δ*rhaBAD*_LD78_ with pKD46-TS	43
KCI1266	As MG1655 plus Δ*tcyP1* Δ*tcyJ1*	Lab collection
KCI1945	MG1655 plus Δ*tcyP1* Δ*tcyJ1* Δ*lacZ1*::*cat* attλ[pSJ501::*cyuR*′*-lacZ*^+^]Cm^r^	This work
KCI1946	MG1655 plus Δ*tcyP1* Δ*tcyJ1* Δ*lacZ1*::*cat* Δ*crp1*::Km attλ[pSJ501::*cyuR*′*-lacZ*^+^]Cm^r^	This work
SR011	As BL21 F^−^ *ompT hsdS*_B_(r_B_^−^ m_B_^−^) *gal dcm* λ(DE3) Δ*katG* Δ*katE*	Lab collection
SSK167	As MG1655 plus *tetRAp-tcyP*	Lab collection
SSK195	As MG1655 plus Δ*tcyP1* Δ*tcyJ1* Δ*cyuA727*::Km	Lab collection
ZYD15	As MG1655 plus Δ*tcyP1* Δ*tcyJ1* Δ*cyuP728*	This work
ZYD154	As MG1655 plus Δ*tcyP1* Δ*tcyJ1* Δ*cyuP728* Δ*lacZ1*::*cam* attλ[pSJ501::*cyuP*′*-lacZ*^+^]	
ZYD156	As MG1655 plus Δ*tcyP1* Δ*tcyJ1* Δ*lacZ1*::*cat* attλ[pSJ501::*cyuP*′*-lacZ*^+^]	This work
ZYD177	As MG1655 plus Δ*tcyP1* Δ*tcyJ1* Δ*lacZ1*::*cat* attλ[pSJ501::*cyuP*′*-lacZ*^+^] Δ*cyuR733*::Km	This work
ZYD229	As SR011 with pET16b-*cyuA* and pDB1282-Isc	This work
ZYD236	As MG1655 plus Δ*tcyP1* Δ*tcyJ1* Δ*lacZ1*::*cat* attλ[pSJ501::*cyuP*′*-lacZ*^+^] Δ*cyuA727*::Km	This work
ZYD238	As MG1655 plus Δ*tcyP1* Δ*tcyJ1* Δ*cyuP728* Δ*cyuA1*::*cat*	This work
ZYD254	As MG1655 plus Δ*tcyP1* Δ*tcyJ1* Δ*cycA757* Δ*brnQ765* Δ*yaaJ727* Δ*liv*(*KMGHF*)*1* attλ[pSJ501::*cyuP*′*-lacZ*^+^]∼*cat*	This work
ZYD256	As MG1655 plus Δ*tcyP1* Δ*tcyJ1* Δ*cyuP728* Δ*cycA757* Δ*brnQ765* Δ*yaaJ727* Δ*liv*(*KMGHF*)*1* attλ[pSJ501::*cyuP*′*-lacZ*^+^]∼*cat*	This work
ZYD266	As MG1655 plus Δ*tcyP1* Δ*tcyJ1* Δ*lacZ1*::*cat* attλ[pSJ501::*cyuP*′*-lacZ*^+^] Δ*crp1*::Km	This work
ZYD277	As MG1655 plus Δ*lacZ1*::*cat* attλ[pSJ501::*cyuP*′*-lacZ*^+^]	This work
ZYD285	As MG1655 *tetRAp-tcyP* Δ*cyuA1*::*cat*	This work
ZYD286	As MG1655 plus Δ*tcyP1* Δ*tcyJ1* Δ*cyuP728 gal*::Tn*10* with pWKS30	This work
ZYD288	As MG1655 plus Δ*tcyP1* Δ*tcyJ1* Δ*cyuP728 gal*::Tn*10* with pWKS30-*cyuP*	This work
ZYD290	As MG1655 plus Δ*tcyP1* Δ*tcyJ1*::*cat* with pWKS30	This work
ZYD292	As MG1655 plus Δ*tcyP1* Δ*tcyJ1*::*cat* with pWKS30-*cyuP*	This work

Plasmids		
pCP20	FLP expression plasmid; Amp^r^; temp-sensitive replication and FLP synthesis	54
pWKS30	Cloning vector; Amp^r^ (6–8 copies per cell)	55
pWKS30-*cyuP*	*cyuP* gene, including 500 bp upstream of the ORF, inserted into pWKS30/KpnI-BamHI; Amp^r^	This work
pET16b	Overexpression vector; T7 promoter and *lac* operator; His_10_ tag; Amp^r^	Novagen
pET16b-*cyuA*	ORF of *cyuA* gene inserted into pET16b/NdeI-BamHI; Amp^r^	This work
pDB1282-*isc*	Expresses proteins including IscS, IcsU, IscA, IscX, HscB, HscA, and Fdx; l-(+)-arabinose-inducible promoter; Kam^r^	56
pKD3	Template plasmid; Amp^r^; *cat* flanked by FRT sites	43
*pKD4*	Template plasmid; Ampr; *kan* flanked by FRT sites	43
pKD46	λ Red recombinase (γ, β, and *exo*) expression plasmid; Amp^r^; l-(+)-arabinose-inducible expression; temp-sensitive replication	43
pSJ501	pAH125 (*lacZ* transcriptional fusion vector) derivative in which the Kam resistance marker has been replaced with a *cat* gene	44; lab collection

a FRT, FLP recombination target.

**TABLE 2:** PCR primers

Purpose	Sequence^*a*^	Relevant plasmid and/or strain
*cyuP-lacZ* transcriptional fusion	F, 5′-AAGTAACTGCAGCAGGGTTAACTGAAC	pSJ130, DH5α *pir*^+^/pSJ501
	R, 5′-AATCAAGGTACCCATAATTTCTCGCTC	

pET16b-*cyuA* overexpression vector	F, 5′-GGCCCATATGATGTTTGATTCGACTTTAAATC	pET16b
	R, 5′-TACCGGATCCTTATCTGGCCTTGC	

pWKS30-*cyuP* with native promoter	F, 5′-ATAGGTACCCTGCGATCCGGTTGCCG	pWKS30
	R, 5′-ATAGGATCCATTAAAAACCTTAAAATTTCAGG	

*cyuA* deletion	F, 5′-CGTTCCTGGCATTTTCTTGATTTACCTGAAATTTTAAGGTTTTTAATATGTGTAGGCTGGAGCTGCTTCG	pKD3
	R, 5′-CATCCGGCACGATCCCCAAAACCTGGCGTTTATCTGGCCTTGCTCGCCATCATATGAATATCCTCCTTAG	

*crp* deletion	F, 5′-GGCGTTATCTGGCTCTGGAGAAAGCTTATAACAGAGGATAACCGCGCATGTGTAGGCTGGAGCTGCTTCG	pKD4
	R, 5′-CTACCAGGTAACGCGCCACTCCGACGGGATTAACGAGTGCCGTAAACGACCATATGAATATCCTCCTTAG	

*cyuR-lacZ* transcriptional fusion	F, 5′-AAGTAAGTCGACACGCTTGCAGATCCAGCTAT	pSJ130, DH5α *pir*^+^/pSJ501
	R, 5′-AATCAAGGTACCCATAGCCCTTCCACAGAGAA	

a F, forward; R, reverse.

### Growth experiments with cysteine as the sole carbon or nitrogen source.

Unless otherwise specified, cells were grown overnight in anoxic minimal medium at 37°C in the anaerobic chamber. For experiments testing cysteine as the carbon source, 22 mM sodium pyruvate and 7.5 mM ammonium sulfate were added to the culture grown overnight and preculture medium. For experiments testing cysteine as the nitrogen source, 0.2% glucose and 1 mM glutamine were added to the culture grown overnight and preculture medium. The cultures grown overnight were then diluted in the same anoxic medium to an OD_600_ of 0.003 and incubated until the cell density reached approximately 0.1 to 0.2, ensuring that cells were fully in exponential phase. During this preculture period, 1 mM cysteine was added to induce *cyuPA*. Cells were washed twice with 10 mL of either room-temperature nitrogen-free medium or carbon-free medium. The washed cells were then diluted to an OD_600_ of 0.007 in 37°C anoxic growth medium that lacked either carbon or nitrogen. Cysteine was then provided as the sole carbon or nitrogen source. The optical densities of the cells were monitored over time.

Growth with 0.5 mM cystine as the sole nitrogen source was conducted in both oxic and anoxic minimal A media. Cells were precultured in minimal glucose medium with 1 mM glutamine as the sole nitrogen source, followed by 2 washes with nitrogen-free medium. They were then suspended in nitrogen-free medium to which 0.5 mM cystine had been added, along with 8 μg/mL tetracycline. The purpose of the tetracycline was to induce the TcyP cystine importer: because the TcyP cystine importer is normally turned off when cystine is available (regulated by CysB) (2, 45), a tetracycline-controllable promoter was constructed in front of *tcyP* (46), and thus, cells could continuously import cystine for a nitrogen source. Tetracycline (8 μg/mL) was provided at a concentration that induced *tcyP* but did not slow down cell growth. Growth was monitored at the OD_600_.

### Analysis of gene expression.

To avoid cysteine oxidation, the expression of the *cyuP-lacZ* and *cyuR-lacZ* fusions was determined under anoxic conditions. Cultures (25 mL) were inoculated from samples grown overnight to an OD_600_ of 0.003 in anoxic minimal medium or LB. Depending on the experiment, various carbon and nitrogen sources were added. Cells were harvested at an OD_600_ of ∼0.1 to 0.2.

The expression of *lacZ* fusions was evaluated by β-galactosidase assays (42, 47). Twenty-milliliter cell cultures were washed with ice-cold 50 mM Tris-HCl (pH 7.4), resuspended in 2 mL of the same buffer, and lysed by a French press. The cell lysates were further centrifuged for 20 min at 12,000 rpm to remove debris prior to assays. The absorbance was monitored at 420 nm for 10 min. Total protein was measured using a Coomassie protein assay (Thermo Scientific), with ovalbumin as the protein standard.

### CyuA anoxic purification and activation.

The E. coli *cyuA* structural gene was amplified by PCR and inserted into the pET16b vector. pET16b provides an N-terminal poly-His linkage to the cloned ORF. After ligation, pET16b-*cyuA* and pDB1282-*isc* were both transformed into SR011 for protein purification. The resulting strain was named ZYD228. SR011 is a modified strain of BL21(DE3), which has *katG katE* deleted. The reason for the *katG katE* deletion is to avoid enzyme copurification of catalase, which would affect our ability to detect the enzyme’s sensitivity to H_2_O_2_. The pDB1282 construct confers kanamycin resistance and contains an E. coli *isc* operon for Fe-S cluster assembly machinery. The operon is cloned behind an l-(+)-arabinose-inducible promoter (48, 49).

A single colony (ZYD228) was inoculated into 5 mL LB with 40 μg/mL kanamycin and 100 μg/mL ampicillin at 37°C in a roller drum. The enriched culture was then diluted down to an OD_600_ of 0.025 in a large volume (e.g., 800 mL LB) containing 40 μg/mL kanamycin and 100 μg/mL ampicillin at 37°C in a shaker. At an OD_600_ of 0.3, 0.2% l-(+)-arabinose was added. Cells were grown at 37°C until the OD_600_ reached 0.6 to 0.8. The culture was kept on ice for 10 min. Next, 0.1 mM IPTG and 50 μM ferrous ammonium sulfate were added. The culture continued with shaking at room temperature for 16 to 18 h. The culture was then washed and resuspended in 25 mL Tris (pH 7.4) buffer for lysis by a French press. The extract was quickly moved into the anaerobic chamber. Since a CyuA homolog was suggested to be oxygen sensitive (3), the subsequent protein purification process was completed in the anaerobic chamber. A His GraviTrap column was equilibrated with anaerobic 50 mM Tris buffer (pH 7.6). The extract was run through the His GraviTrap column to bind His-linked CyuA. Imidazole solutions were prechilled on ice. Five milliliters of 5 mM, 10 mL of 10 mM, 5 mL of 20 mM, and 10 mL of 60 mM imidazole–150 mM NaCl–50 mM Tris (pH 7.6) (prechilled) were used to wash the column. Five milliliters of 50 mM Tris (pH 7.6)–150 mM imidazole–150 mM NaCl and 5 mL of 50 mM Tris (pH 7.6)–200 mM imidazole–150 mM NaCl (prechilled) were used to elute CyuA. These elution fractions were combined into 10 mL, and imidazole was removed by centrifugation in 15-mL Amicon Ultra centrifugal filters (10K) with 50 mM Tris (pH 7.6)–150 mM NaCl twice, at 12°C, with a 2-mL volume left. Glycerol was added to the eluted solution to a final concentration of 20%. The obtained CyuA protein concentration was approximately 80 μM. The protein was colored, consistent with an iron-sulfur cluster protein. Aliquots (100 μL) were frozen in dry ice and stored at −80°C. Based on the band intensity from SDS-PAGE gels, the protein is about 95% pure. The size of CyuA containing 10×His tags is approximately 47 kDa.

The purified CyuA did not display activity according to the coupled lactic dehydrogenase assay (below). However, the enzyme could be activated by incubation with 0.5 mM ferrous ammonium iron and 5 mM DTT in 50 mM Tris (pH 7.6) for 30 min at room temperature. The CyuA protein concentration used for activation ranged from 4 to 8 μM.

### Assay of pyruvate formation by CyuA.

When CyuA breaks down cysteine into pyruvate, lactic dehydrogenase will convert pyruvate to lactic acid as it oxidizes NADH to NAD^+^. The disappearance of NADH can be monitored by a spectrometer. Purified CyuA was assayed at room temperature by dilution into a reaction mixture containing 50 mM Tris buffer (pH 7.6), 100 μM NADH, 5 U lactic dehydrogenase, and various concentrations of cysteine in a final reaction volume of 500 μL in a sealed anaerobic cuvette. The absorbance was monitored at 340 nm for 8 min.

d-Cysteine, l-serine, d-serine, l-leucine, l-alanine, *N*-acetylcysteine, homocysteine, thioglycolic acid, glutathione, β-mercaptoethanol, and cysteamine were used at 2 mM as a 10-fold excess of competitors of 0.2 mM l-cysteine for CyuA. Thiol-containing competitors were dissolved in double-deionized water and prepared fresh in the anaerobic chamber to prevent oxidation and H_2_O_2_ production.

H_2_O_2_, l-cysteine, *N*-acetylcysteine, cysteamine, β-mercaptoethanol, homocysteine, and d-cysteine were checked to see if they are inhibitors of lactic dehydrogenase. Pyruvate (100 μM), 100 μM NADH, 2 mM “inhibitors” or 1 mM H_2_O_2_, 50 mM Tris buffer (pH 7.6), and 0.05 U lactic dehydrogenase were added to the reaction mixture to make a final volume of 500 μL. The reaction mixture was premixed in an Eppendorf tube prior to transfer to the cuvette. The absorbance was monitored at 340 nm for 10 min.

To test the sensitivity of purified CyuA to oxygen, superoxide dismutase (60 U/mL) and catalase (60 U/mL) were added to prevent the accumulation of H_2_O_2_ and superoxide. DTPA (100 μM) was added to prevent a possible Fenton reaction. After the assay was run for 2 min, the cuvette lid was opened, and air was introduced by pipetting the reaction mixture down the walls of the cuvette. The rate of NADH oxidation was measured before and after aeration.

### [^14^C]cysteine transport assay.

Cultures grown overnight (5 mL) in anoxic pyruvate-ammonium medium were diluted to an OD_600_ of 0.003 in the same medium. After 2 doublings (OD_600_ of ∼0.012), 10 mM cysteine was added to fully induce *cyuP*. In some experiments, the carbon source was changed to 0.2% glucose, and the nitrogen source was changed to 1 mM glutamine. Regardless, cells were harvested at an OD_600_ of ∼0.08 to 0.10, centrifuged twice at 16°C to wash away the cold cysteine, and resuspended in 10 mL of glucose-ammonia medium. The final resuspension was adjusted to an OD_600_ of 0.5. Cells were incubated for 5 min with 80 μg/mL chloramphenicol to block protein synthesis. l-[1,2,1′,2′-^14^C]cystine was mixed with cold cystine in glucose-ammonium medium to obtain the desired specific activity and a stock concentration of 100 μM. The cystine was freshly reduced to cysteine by DTT (10 mM) at 37°C for more than 30 min, while DTPA (1 mM) was included to suppress cysteine oxidation. The final [^14^C]cysteine cocktail in glucose-ammonia medium was added to cells at 37°C to establish a final concentration of 20 μM (or various cysteine concentrations for *K_M_* measurements) and, depending on the experiment, 3 to 40 mCi/mmol cysteine. The cocktail mix (50 μL) was then aliquoted into 450 μL of prewarmed (37°C) chloramphenicol-treated cells. At intervals (5 s, 15 s, 45 s, and 60 s), the new mixture (cocktail plus cells) was aliquoted (70 μL) onto a presoaked filter on a filtration manifold and washed three times with 2.5 mL of wash buffer (100 mM Tris [pH 7.4], 0.15 M NaCl, and 0.5 mM MgCl_2_ at room temperature). Wash times were ca. 5 s; the delay applied to all time points and was remedied by using the slope of the time course. Filters were dried by a heat lamp, transferred to vials containing 5 mL scintillation fluid, and analyzed by scintillation counting. In some experiments, other unlabeled amino acids at a 10-fold excess (200 μM) were added to cells prior to the addition of cysteine so that their ability to inhibit cysteine import could be appraised. The intracellular concentrations of cysteine were calculated using the fact that 1 mL × 1 OD_600_ of E. coli contains 0.5 μL of cytoplasm (50). Therefore, from the specific activities of the radiolabeled amino acids and the amount of cell-associated radioactivity, the cytoplasmic concentrations of the imported amino acids were derived, and the import rates were presented as millimolar cytoplasmic cysteine per unit time.

### Mixed-culture growth experiment.

KCI1266 (*cyuA*^+^) and SSK195 (Δ*cyuA*, conferring kanamycin resistance) were used for mixed-culture experiments in anoxic glucose-ammonium medium. To determine whether *cyuA*^+^ cells could protect Δ*cyuA* cells by scavenging extracellular cysteine, both cultures grown overnight were diluted to an OD_600_ of 0.0125 to grow for 3 doublings until they reached an OD_600_ of 0.2. Cells were then back-diluted to a low OD to monitor growth. The initial inoculation OD_600_ of *cyuA*^+^ and Δ*cyuA* strains was at a 4:1 ratio (OD of 0.004/0.001). The *cyuA*^+^ and Δ*cyuA* strains were also incubated in separate cultures as controls. Cysteine (5 mM) was added to each culture. To keep cysteine reduced, cultures were grown anaerobically.

For nitrogen cross-feeding experiments, cells were grown in anaerobic minimal nitrogen-free medium. The starting absorbance ratio of KCI1266 to SSK195 was 4:1. In the preculture, glucose and glutamine (1 mM) were provided as the carbon and nitrogen sources. Once cells reached an OD_600_ of 0.2, they were washed and resuspended in glucose medium with 1 mM cysteine as the sole nitrogen source. Both the total absorbance and strain-specific CFU were tracked.

For both experiments, time points were taken every 2 h. The number of CFU per milliliter was determined by serial dilutions in LB. The dilutions were then mixed with 2.5 mL top agar (0.8%) (at 50°C) and spread onto LB (to quantify total cells) and LB-kanamycin (40 μg/mL) (to quantify Δ*cyuA* mutants) plates. Plates were incubated for 18 h before counting. Top agar with or without the addition of antibiotics did not change the CFU.

### CyuP^+/−^ competition experiment.

The goal was to see whether CyuP could provide any growth advantage for the cells grown on moderate levels of cysteine. ZYD286 (Δ*cyuP*, tetracycline resistant, with pWKS30) was mixed with ZYD290 (*cyuP*, chloramphenicol resistant, with pWKS30); ZYD288 (Δ*cyuP*, tetracycline resistant, with pWKS30-*cyuP*) was mixed with ZYD292 (*cyuP*, chloramphenicol resistant, with pWKS30-*cyuP*). The antibiotic resistance gene from the Δ*cyuP* strain was flipped out by pCP20 to avoid downstream polar effects on *cyuA*. The *cyuP* gene was provided on a low-copy-number plasmid (pWKS30). Each strain was precultured to log phase in pyruvate-ammonium medium and glucose-glutamine medium, each containing 1 mM cysteine to induce the *cyuPA* operon. The competing strains were then mixed at a 1:1 ratio, starting at an OD_600_ of 0.003. The glucose-glutamine-precultured cells were cultured in glucose medium containing 0.2 mM cysteine as the sole nitrogen source, and the pyruvate-ammonium-precultured cells were cultured in medium containing 1 mM cysteine as the sole carbon source with ammonium sulfate as the nitrogen source. Growth was monitored by tracking the OD_600_. Once growth reached a plateau from the exhaustion of cysteine, some cells were plated onto antibiotic plates, and the culture was back-diluted 100-fold to start another round of growth. The numbers of CFU per milliliter were determined by serial dilutions in LB. The dilutions were then mixed with 2.5 mL top agar (0.8% agar) (at 50°C) and spread onto LB containing chloramphenicol (20 μg/mL) with ampicillin (100 μg/mL) (to quantify *cyuP^+^* cells) and LB containing tetracycline (8 μg/mL) with ampicillin (100 μg/mL) (to quantify Δ*cyuP* cells) plates. Plates were incubated aerobically for 18 h before counting.

### DTNB cysteine detection assay.

A total of 50 mM DTNB, also known as the Ellman’s reagent (51), was prepared in ethanol. At time intervals, 995 μL of culture medium that contains cysteine (at most a 100 μM final concentration for accurate measurement) was added to 5 μL of 50 mM DTNB, followed by a quick spin to pellet the cells. Depending on the cysteine concentration, dilutions in 50 mM Tris buffer (pH 7.6) were necessary to obtain measurable data. The absorbance was measured at 412 nm. The cysteine concentration was determined by the colored product formed from DTNB reacting with thiol (1:1 ratio), 2-nitro-5-thiobenzoate, with an extinction coefficient of 14,150 M^−1^ cm^−1^.

### Lead acetate strip for sulfide detection.

All steps were performed in an anaerobic chamber with anoxic materials. Cells with different mutations were grown in minimal medium with pyruvate (22 mM) as the carbon source and ammonium to an OD_600_ ∼0.1. The cells (100 μL) were then top spread onto plates of the same composition but that also contained 0.2 mM isoleucine to avoid cysteine toxicity. Sterile discs (6 mm) were soaked in 1 M cysteine and placed in the middle of the plate. Lead acetate strips were taped inside the lid to detect sulfide production. Plates were wrapped with parafilm. Pictures were scanned every 6 h. The lids were removed while pictures were taken.

### Direct sulfide determination.

Sulfide was detected colorimetrically by incorporation into methylene blue in the presence of *N*,*N*-diethyl-*p*-phenylenediamine (DPD) (52). During cell growth on cysteine, samples were directly taken from the growing culture, followed by a quick spin. The supernatant (800 μL) was mixed with 100 μL 20 mM DPD (in 6 N HCl) and 100 μL 30 mM FeCl_3_ (in 1.2 N HCl). The reaction was continued in the dark for 30 min at room temperature. The absorbance was then measured at 670 nm. We found that the amount of cysteine affects sulfide detection. Zero, 1 mM, 2 mM, 5 mM, and 10 mM cysteine were tested. The more cysteine in the reaction, the lower the sulfide absorbance that was displayed at 670 nm. This is possibly because cysteine in large amounts is more capable of chelating iron, which is crucial for the methylene blue reaction. Thus, growth with 1 mM cysteine was chosen for sulfide determinations.

10.1128/mBio.02965-21.1 TEXT S1 (A) Comparison of cysteine import rates to cell demand for nitrogen and carbon. (B) Calculation of the half-time for the diffusion of free ammonia from the cell. Download Text S1, DOCX file, 0.02 MB. Copyright © 2022 Zhou and Imlay. 2022 Zhou and Imlay. https://creativecommons.org/licenses/by/4.0/ This content is distributed under the terms of the Creative Commons Attribution 4.0 International license.

10.1128/mBio.02965-21.8 FIG S7 Cysteine consumption is driven primarily by CyuA. (A) Utilization of cysteine as a nitrogen source relies mainly on CyuA. One millimolar cysteine was supplied as the nitrogen source. The strains used are KCI1266 and SSK195. (B) Cysteine utilization as a carbon source also relies on CyuA. The medium contained ammonium with 10 mM cysteine as the carbon source. The strains used are KCI1266 and SSK195. Download FIG S7, TIF file, 0.9 MB. Copyright © 2022 Zhou and Imlay. 2022 Zhou and Imlay. https://creativecommons.org/licenses/by/4.0/ This content is distributed under the terms of the Creative Commons Attribution 4.0 International license.

## References

[1] Korshunov S, Imlay KRC, Imlay JA. 2020. Cystine import is a valuable but risky process whose hazards *Escherichia coli* minimizes by inducing a cysteine exporter. Mol Microbiol 113:22–39. doi:10.1111/mmi.14403. 31612555 PMC7007315

[2] Imlay KRC, Korshunov S, Imlay JA. 2015. The physiological roles and adverse effects of the two cystine importers of *Escherichia coli*. J Bacteriol 197:3629–3644. doi:10.1128/JB.00277-15. 26350134 PMC4626903

[3] Tchong S-I, Xu H, White RH. 2005. l-Cysteine desulfidase: an [4Fe-4S] enzyme isolated from *Methanocaldococcus jannaschii* that catalyzes the breakdown of l-cysteine into pyruvate, ammonia, and sulfide. Biochemistry 44:1659–1670. doi:10.1021/bi0484769. 15683250

[4] Mendez J, Reimundo P, Perez-Pascual D, Navais R, Gomez E, Guijarro JA. 2011. A novel *cdsAB* operon is involved in the uptake of l-cysteine and participates in the pathogenesis of *Yersinia ruckeri*. J Bacteriol 193:944–951. doi:10.1128/JB.01058-10. 21169490 PMC3028680

[5] Shimada T, Tanaka K, Ishihama A. 2016. Transcription factor DecR (YbaO) controls detoxification of L-cysteine in *Escherichia coli*. Microbiology (Reading) 162:1698–1707. doi:10.1099/mic.0.000337. 27435271

[6] Loddeke M, Schneider B, Oguri T, Mehta I, Xuan ZY, Reitzer L. 2017. Anaerobic cysteine degradation and potential metabolic coordination in *Salmonella enterica* and *Escherichia coli*. J Bacteriol 199:e00117-17. doi:10.1128/JB.00117-17. 28607157 PMC5527379

[7] Oguri T, Schneider B, Reitzer L. 2012. Cysteine catabolism and cysteine desulfhydrase (CdsH/STM0458) in *Salmonella enterica* serovar Typhimurium. J Bacteriol 194:4366–4376. doi:10.1128/JB.00729-12. 22685283 PMC3416216

[8] Harris CL. 1981. Cysteine and growth inhibition of *Escherichia coli*: threonine deaminase as the target enzyme. J Bacteriol 145:1031–1035. doi:10.1128/jb.145.2.1031-1035.1981. 7007336 PMC217214

[9] Sorensen MA, Pedersen S. 1991. Cysteine, even in low concentrations, induces transient amino acid starvation in *Escherichia coli*. J Bacteriol 173:5244–5246. doi:10.1128/jb.173.16.5244-5246.1991. 1907268 PMC208221

[10] Kari C, Nagy Z, Kovacs P, Hernadi F. 1971. Mechanism of the growth inhibitory effect of cysteine on *Escherichia coli*. J Gen Microbiol 68:349–356. doi:10.1099/00221287-68-3-349. 4944587

[11] Park S, Imlay JA. 2003. High levels of intracellular cysteine promote oxidative DNA damage by driving the Fenton reaction. J Bacteriol 185:1942–1950. doi:10.1128/JB.185.6.1942-1950.2003. 12618458 PMC150142

[12] Zhou Y, Imlay JA. 2020. *Escherichia coli* K-12 lacks a high-affinity assimilatory cysteine importer. mBio 11:e01073-20. doi:10.1128/mBio.01073-20. 32518189 PMC7373191

[13] Strecker A, Schubert C, Zedler S, Steinmetz P, Unden G. 2018. DcuA of aerobically grown *Escherichia coli* serves as a nitrogen shuttle (L-aspartate/fumarate) for nitrogen uptake. Mol Microbiol 109:801–811. doi:10.1111/mmi.14074. 29995997

[14] Burrous SE, Demoss RD. 1963. Studies on tryptophan permease in *Escherichia coli*. Biochim Biophys Acta 73:623–637. doi:10.1016/0006-3002(63)90332-9. 14074136

[15] Flint DH, Tuminello JF, Emptage MH. 1993. The inactivation of Fe-S cluster containing hydro-lyases by superoxide. J Biol Chem 268:22369–22376. doi:10.1016/S0021-9258(18)41538-4. 8226748

[16] Varghese S, Tang Y, Imlay JA. 2003. Contrasting sensitivities of *Escherichia coli* aconitases A and B to oxidation and iron depletion. J Bacteriol 185:221–230. doi:10.1128/JB.185.1.221-230.2003. 12486059 PMC141816

[17] Zhang CM, Christian T, Newberry KJ, Perona JJ, Hou YM. 2003. Zinc-mediated amino acid discrimination in cysteinyl-tRNA synthetase. J Mol Biol 327:911–917. doi:10.1016/s0022-2836(03)00241-9. 12662918

[18] Smith EA, Macfarlane GT. 1998. Enumeration of amino acid fermenting bacteria in the human large intestine: effects of pH and starch on peptide metabolism and dissimilation of amino acids. FEMS Microbiol Ecol 25:355–368. doi:10.1111/j.1574-6941.1998.tb00487.x.

[19] Bertin Y, Segura A, Jubelin G, Duniere L, Durand A, Forano E. 2018. Aspartate metabolism is involved in the maintenance of enterohaemorrhagic *Escherichia coli* O157:H7 in bovine intestinal content. Environ Microbiol 20:4473–4485. doi:10.1111/1462-2920.14380. 30109758

[20] Yamamoto Y, Nakanishi Y, Murakami S, Aw W, Tsukimi T, Nozu R, Ueno M, Hioki K, Nakahigashi K, Hirayama A, Sugimoto M, Soga T, Ito M, Tomita M, Fukuda S. 2018. A metabolomic-based evaluation of the role of commensal microbiota throughout the gastrointestinal tract in mice. Microorganisms 6:101. doi:10.3390/microorganisms6040101. PMC6313407 30274293

[21] Awano N, Wada M, Mori H, Nakamori S, Takagi H. 2005. Identification and functional analysis of *Escherichia coli* cysteine desulfhydrases. Appl Environ Microbiol 71:4149–4152. doi:10.1128/AEM.71.7.4149-4152.2005. 16000837 PMC1169034

[22] Mironov A, Seregina T, Nagornykh M, Luhachack LG, Korolkova N, Lopes LE, Kotova V, Zavilgelsky G, Shakulov R, Shatalin K, Nudler E. 2017. Mechanism of H_2_S-mediated protection against oxidative stress in *Escherichia coli*. Proc Natl Acad Sci USA 114:6022–6027. doi:10.1073/pnas.1703576114. 28533366 PMC5468659

[23] Zdych E, Peist R, Reidl J, Boos W. 1995. MalY of *Escherichia coli* is an enzyme with the activity of a β-C-S lyase (cystathionase). J Bacteriol 177:5035–5039. doi:10.1128/jb.177.17.5035-5039.1995. 7665481 PMC177281

[24] Reitzer LJ, Magasanik B. 1983. Isolation of the nitrogen assimilation regulator NR(I), the product of the *glnG* gene of *Escherichia coli*. Proc Natl Acad Sci USA 80:5554–5558. doi:10.1073/pnas.80.18.5554. 16593366 PMC384296

[25] Soupene E, He L, Yan D, Kustu S. 1998. Ammonia acquisition in enteric bacteria: physiological role of the ammonium/methylammonium transport B (AmtB) protein. Proc Natl Acad Sci USA 95:7030–7034. doi:10.1073/pnas.95.12.7030. 9618533 PMC22728

[26] Gorke B, Stulke J. 2008. Carbon catabolite repression in bacteria: many ways to make the most out of nutrients. Nat Rev Microbiol 6:613–624. doi:10.1038/nrmicro1932. 18628769

[27] Deutscher J. 2008. The mechanisms of carbon catabolite repression in bacteria. Curr Opin Microbiol 11:87–93. doi:10.1016/j.mib.2008.02.007. 18359269

[28] Fic E, Bonarek P, Gorecki A, Kedracka-Krok S, Mikolajczak J, Polit A, Tworzydlo M, Dziedzicka-Wasylewska M, Wasylewski Z. 2009. cAMP receptor protein from *Escherichia coli* as a model of signal transduction in proteins—a review. J Mol Microbiol Biotechnol 17:1–11. doi:10.1159/000178014. 19033675

[29] Kolb A, Busby S, Buc H, Garges S, Adhya S. 1993. Transcriptional regulation by cAMP and its receptor protein. Annu Rev Biochem 62:749–795. doi:10.1146/annurev.bi.62.070193.003533. 8394684

[30] Lu Z, Sethu R, Imlay JA. 2018. Endogenous superoxide is a key effector of the oxygen sensitivity of a model obligate anaerobe. Proc Natl Acad Sci USA 115:e3266–e3275. doi:10.1073/pnas.1800120115. 29559534 PMC5889672

[31] Burman JD, Harris RL, Hauton KA, Lawson DM, Sawers RG. 2004. The iron-sulfur cluster in the L-serine dehydratase TdcG from *Escherichia coli* is required for enzyme activity. FEBS Lett 576:442–444. doi:10.1016/j.febslet.2004.09.058. 15498577

[32] Baptist EW, Kredich NM. 1977. Regulation of l-cystine transport in *Salmonella typhimurium*. J Bacteriol 131:111–118. doi:10.1128/jb.131.1.111-118.1977. 326753 PMC235398

[33] Reitzer L. 25 July 2005. Catabolism of amino acids and related compounds. EcoSal Plus 2005 10.1128/ecosalplus.3.4.7. 26443507

[34] Gottschalk G. 1986. Bacterial metabolism, 2nd ed. Springer-Verlag, New York, NY.

[35] Nonaka G, Takumi K. 2017. Cysteine degradation gene *yhaM*, encoding cysteine desulfidase, serves as a genetic engineering target to improve cysteine production in *Escherichia coli*. AMB Expr 7:90. doi:10.1186/s13568-017-0389-y. PMC5423876 28488255

[36] Kredich NM, Foote LJ, Keenan BS. 1973. The stoichiometry and kinetics of the inducible cysteine desulfhydrase from *Salmonella typhimurium*. J Biol Chem 248:6187–6196. doi:10.1016/S0021-9258(19)43526-6. 4580051

[37] Kredich NM, Keenan BS, Foote LJ. 1972. The purification and subunit structure of cysteine desulfhydrase from *Salmonella typhimurium*. J Biol Chem 247:7157–7162. doi:10.1016/S0021-9258(19)44608-5. 4565078

[38] Deplancke B, Finster K, Graham WV, Collier CT, Thurmond JE, Gaskins HR. 2003. Gastrointestinal and microbial responses to sulfate-supplemented drinking water in mice. Exp Biol Med (Maywood) 228:424–433. doi:10.1177/153537020322800413. 12671187

[39] Sabrialabe S, Yang JG, Yariv E, Ben-Tal N, Lewinson O. 2020. Substrate recognition and ATPase activity of the *E. coli* cysteine/cystine ABC transporter YecSC-FliY. J Biol Chem 295:5245–5256. doi:10.1074/jbc.RA119.012063. 32144203 PMC7170509

[40] Muller A, Thomas GH, Horler R, Brannigan JA, Blagova E, Levdikov VM, Fogg JJ, Wilson KS, Wilkinson AJ. 2005. An ATP-binding cassette-type cysteine transporter in *Campylobacter jejuni* inferred from the structure of an extracytoplasmic solute receptor protein. Mol Microbiol 57:143–155. doi:10.1111/j.1365-2958.2005.04691.x. 15948956

[41] Ewann F, Hoffman PS. 2006. Cysteine metabolism in *Legionella pneumophila*: characterization of an l-cystine-utilizing mutant. Appl Environ Microbiol 72:3993–4000. doi:10.1128/AEM.00684-06. 16751507 PMC1489648

[42] Miller JH. 1972. Experiments in molecular genetics. Cold Spring Harbor Laboratory, Cold Spring Harbor, NY.

[43] Datsenko KA, Wanner BL. 2000. One-step inactivation of chromosomal genes in *Escherichia coli* K-12 using PCR products. Proc Natl Acad Sci USA 97:6640–6645. doi:10.1073/pnas.120163297. 10829079 PMC18686

[44] Haldimann A, Wanner BL. 2001. Conditional-replication, integration, excision, and retrieval plasmid-host systems for gene structure-function studies of bacteria. J Bacteriol 183:6384–6393. doi:10.1128/JB.183.21.6384-6393.2001. 11591683 PMC100134

[45] Kredich NM. 1996. Biosynthesis of cysteine, p 514–527. *In* Neidhardt FC, Curtiss R, III, Ingraham JL, Lin ECC, Low KB, Magasanik B, Reznikoff WS, Riley M, Schaechter M, Umbarger HE (ed), Escherichia coli and Salmonella: cellular and molecular biology, 2nd ed, vol 1. ASM Press, Washington, DC.

[46] Merighi M, Ellermeier CD, Slauch JM, Gunn JS. 2005. Resolvase-in vivo expression technology analysis of the *Salmonella enterica* serovar Typhimurium PhoP and PmrA regulons in BALB/c mice. J Bacteriol 187:7407–7416. doi:10.1128/JB.187.21.7407-7416.2005. 16237024 PMC1272988

[47] Casadaban MJ, Martinezarias A, Shapira SK, Chou J. 1983. Beta-galactosidase gene fusions for analyzing gene expression in *Escherichia coli* and yeast. Methods Enzymol 100:293–308. doi:10.1016/0076-6879(83)00063-4. 6312261

[48] Dos Santos PC, Johnson DC, Ragle BE, Unciuleac MC, Dean DR. 2007. Controlled expression of nif and isc iron-sulfur protein maturation components reveals target specificity and limited functional replacement between the two systems. J Bacteriol 189:2854–2862. doi:10.1128/JB.01734-06. 17237162 PMC1855825

[49] Lanz ND, Grove TL, Gogonea CB, Lee K-H, Krebs C, Booker SJ. 2012. RlmN and AtsB as models for the overproduction and characterization of radical SAM proteins. Methods Enzymol 516:125–152. doi:10.1016/B978-0-12-394291-3.00030-7. 23034227

[50] Imlay JA, Fridovich I. 1991. Assay of metabolic superoxide production in *Escherichia coli*. J Biol Chem 266:6957–6965. doi:10.1016/S0021-9258(20)89596-9. 1849898

[51] Ellman GL. 1959. Tissue sulfhydryl groups. Arch Biochem Biophys 82:70–77. doi:10.1016/0003-9861(59)90090-6. 13650640

[52] Siegel LM. 1965. A direct microdetermination for sulfide. Anal Biochem 11:126–132. doi:10.1016/0003-2697(65)90051-5. 14328633

[53] Neidhardt FC, Curtiss R, III, Ingraham JL, Lin ECC, Low KB, Magasanik B, Reznikoff WS, Riley M, Schaechter M, Umbarger HE (ed). 1996. Escherichia coli and Salmonella: cellular and molecular biology, 2nd ed. ASM Press, Washington, DC.

[54] Cherepanov PP, Wackernagel W. 1995. Gene disruption in *Escherichia coli*: TcR and KmR cassettes with the option of Flp-catalyzed excision of the antibiotic-resistance determinant. Gene 158:9–14. doi:10.1016/0378-1119(95)00193-a. 7789817

[55] Wang RF, Kushner SR. 1991. Construction of versatile low-copy-number vectors for cloning, sequencing and gene expression in *Escherichia coli*. Gene 100:195–199. doi:10.1016/0378-1119(91)90366-J. 2055470

[56] Zheng L, Cash VL, Flint DH, Dean DR. 1998. Assembly of iron-sulfur clusters. Identification of an *iscSUA-hscBA-fdx* gene cluster from *Azotobacter vinelandii*. J Biol Chem 273:13264–13272. doi:10.1074/jbc.273.21.13264. 9582371

[57] Seaver LC, Imlay JA. 2001. Hydrogen peroxide fluxes and compartmentalization inside growing *Escherichia coli*. J Bacteriol 183:7182–7189. doi:10.1128/JB.183.24.7182-7189.2001. 11717277 PMC95567

[58] Cueto-Rojas HF, Milne N, van Helmond W, Pieterse MM, van Maris AJA, Daran J-M, Wahl SA. 2017. Membrane potential independent transport of NH_3_ in the absence of ammonium permeases in *Saccharomyces cerevisiae*. BMC Syst Biol 11:49. doi:10.1186/s12918-016-0381-1. 28412970 PMC5392931

