# Supplementary material for: Escherichia coli Uses a Dedicated Importer and Desulfidase To Ferment Cysteine
Source: mBio. 2022 Apr 4;13(2):e02965-21. doi: 10.1128/mbio.02965-21 (PMC9040844; doi:10.1128/mbio.02965-21)
Supplement: TEXT S1 [file mbio.02965-21-s0001.docx]

**Supplementary calculations.**

**1. Comparison of cysteine-import rates to cell demand for nitrogen and carbon.**

Fig. 5A shows that 2.6 mM nitrogen is needed to achieve an OD_600_ = 1. Fig. S7 shows that the cysteine consumption is 450 μM over a time period during which the cell density increased by 0.201 OD_600_; the expected cysteine consumption would be 2.6 mM/OD x 0.201 OD = 520 μM, which we regard as reasonable agreement. Similar calculations, under conditions in which cysteine was used as sole carbon source, predicted 4.0 mM cysteine degradation during a period in which 4.4 mM cysteine was eliminated from the growth medium.

If 2.6 mM nitrogen is needed to achieve an OD_600_ = 1, and 1 L of 1 OD contains 0.5 ml total cytoplasm [50], the total nitrogen concentration in the cytoplasm is 5.6 M, which is mainly in the form of proteins and nucleic acids. The doubling time is 144 min (k = 0.0048 min^-1^), and so the required rate of nitrogen influx to support this growth rate is 0.0048 min^-1^ x 5.6 M nitrogen =27 mM/min. This is the rate at which cysteine must be imported and degraded.

The analogous carbon-demand measurements indicated that cells need to consume 43 mM cysteine to reach OD_600_ = 1. The doubling time is 350 min (k = 0.0020), and one calculates that the necessary rate of carbon influx is 170 mM/min. Both of these rates are substantially below the cysteine-import rates that we measured in transport assays, which were typically 10-20 mM/min. However, during those experiments most the imported cysteine was continuously degraded to formate and acetate, which effuse back out of the cell; thus, the rate of radiolabel accumulation did not represent the total rate of import.

**2. Calculation of the half-time for the diffusion of free ammonia from the cell.**

We sought to calculate how quickly ammonia would diffuse out of an *E. coli* cell were the ammonia not scavenged by glutamine synthetase or glutamate dehydrogenase. The point was to evaluate how efficient those two enzymes must be to capture the ammonia that is released by amino acid deaminases, such as CyuA. Data presented in this paper show that ammonia is trapped inside the cell that produces it, as by-stander bacteria fail to acquire the liberated nitrogen.

In the absence of trapping the steady-state NH_3_ concentration is the balance between its formation and its diffusion out of the cell. The calculation is performed for NH_3_ rather than the ammonium cation, as the latter charged species does not cross membranes.

In the steady-state situation, the rate of NH_3_ production equals the rate of efflux:

V_production_ = V_efflux_

The rate of production of ammonia from cysteine under our growth conditions was shown above to be 27 mM/min, or 0.45 mM/sec. Using cell dimensions measured previously, in minimal medium [50, 57]:

The ammonium production flux in mol/s = (4.5 x 10^-4^ mol/L/sec) x (7 x 10^-16^ L/cell)

= 3.2 x 10^-19^ mol/cell/sec

At cytoplasmic pH (~7.4) the pKa of ammonia (9.26) dictates that only 1.4% is deprotonated, so the NH_3_ production flux = 4.5 x 10^-21^ mol/cell/sec.

The rate of efflux is determined by the internal NH_3_ concentration, the membrane surface area, and the membrane permeability coefficient for NH_3_. That coefficient is 4.8 x 10^-2^ cm/sec [58]. If the cellular cytoplasm is modeled as a simple cylinder of dimensions 0.32 μm radius x 2.2 μm length (consistent with the measured volume), then the membrane surface area = 4.5 x 10^-8^ cm^2^. Then the ammonia efflux

= [NH_3_] x permeability coefficient x surface area

= [NH_3_] x (4.8 x 10^-2^ cm/sec) x (4.5 x 10^-8^ cm^2^/cell) x 10^-3^ L/cm^3^

Thus 4.5 x 10^-21^ mol/cell/sec = [NH_3_] x 2.2 x 10^-12^ L/cell/sec

and steady-state [NH_3_] = 2.0 x 10^-9^ M.

One can calculate the rate constant for efflux by setting NH_3_ production equal to efflux, noting that NH_3_ production = 0.45 mM/sec x 1.4% = 6.3 μM/sec:

6.3 μM/sec = [NH_3_] x k

If [NH_3_] = 2.0 x 10^-9^ M, then k = 3.2 x 10^3^ s^-1^

If d[NH_3_]/dt = [NH_3_] x k, then Ln{[NH_3_]/[NH_3_]_o_}(Burrous & Demoss, 1963) = -kt

and t_1/2_ = 0.2 msec.

Because ammonia is only deprotonated 1.4% of the time, and the ammonium ion should not cross membranes, the half-time for efflux of total ammonia (deprotonated ammonia plus ammonium ion) is 15 msec. This value is an overestimate if the membranes are invaginated, and it suffices to show that the near-quantitative trapping of released ammonium after cysteine desulfidation is impressive. Interestingly, this result also implies that ammonium-trapping will be similarly effective when NH_4_^+^ enters nitrogen-limited cells from the environment: Any incoming ammonium will be captured.

50. Imlay JA, Fridovich I. 1991. Assay of metabolic superoxide production in *Escherichia coli*. J Biol Chem 266:6957-65.

57. Seaver LC, Imlay JA. 2001. Hydrogen peroxide fluxes and compartmentalization inside growing *Escherichia coli*. J Bacteriol 183:7182-7189.

58. Cueto-Rojas HF, Milne N, van Helmond W, Pieterse MM, van Maris AJA, Daran J-M, Wahl SA. 2017. Membrane potential independent transport of NH_3_ in the absence of ammonium permeases in *Saccharomyces cerevisiae*. BMC Syst Biol 11:49.
